# Moiré Mott Correlated Mosaics in Twisted Bilayer 1T-TaS_2_

**DOI:** 10.1021/acs.nanolett.6c01799

**Published:** 2026-08-24

**Authors:** Ana Vera Montoto, Jose L. Lado, Adolfo O. Fumega

**Affiliations:** Department of Applied Physics, 174277 Aalto University, 02150 Espoo, Finland

**Keywords:** van der Waals materials, transition metal dichalcogenides, charge density wave, moiré, Mott insulator

## Abstract

The tunability and twist engineering of van der Waals materials enable the emergence of electronic states not present in individual monolayers. Among them, monolayer 1T-TaS_2_ is a well-known Mott insulating system, whose star-of-David charge density wave reconstruction realizes an emergent triangular lattice of local magnetic moments. Interestingly, in its bulk form, the insulating gap is not correlation-driven but stems from interlayer coupling. Here, we exploit the stacking-dependent nature of the insulating gap to show that, in twisted 1T-TaS_2_ bilayers, the spatially dependent competition between many-body and single-particle gaps creates Mott–trivial mosaic superlattices, featuring regions with local magnetic moments and nonmagnetic insulating regions. We further demonstrate the tunability of the mosaic correlated state with an interlayer bias, giving rise to controllable charge transfer and quenching of correlations. Our results establish twisted 1T-TaS_2_ as a flexible platform to engineer mixed spatially modulated correlated insulating phases, arising from the moiré profile.

## Graphical abstract

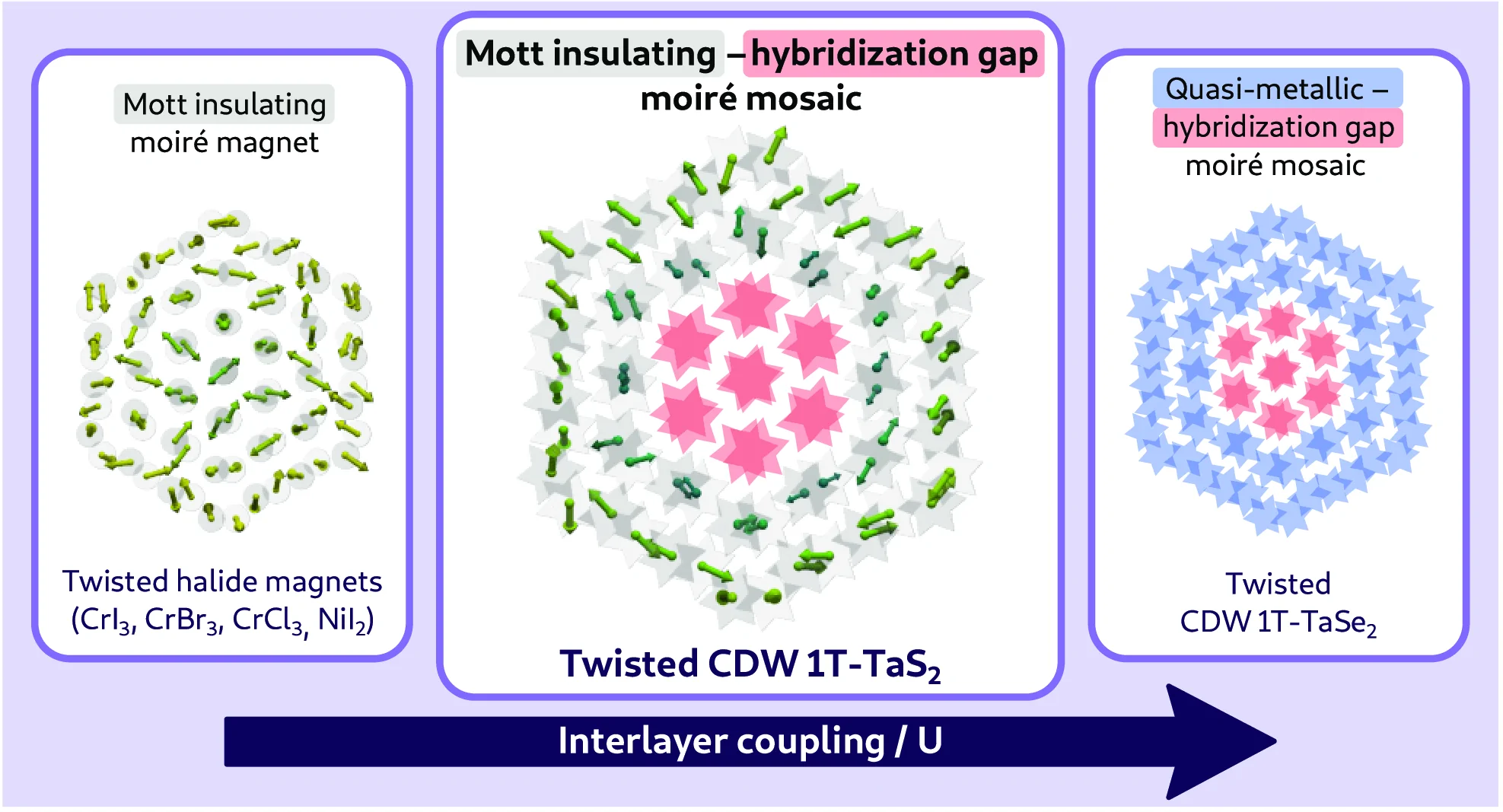

Twisted van der Waals heterostructures have risen as a widely flexible platform to engineer new forms of quantum matter.^1^ The twist degree of freedom produces new superlattices, whose emergent physics arises from the spatially dependent coupling between layers. This strategy has been demonstrated using two-dimensional semimetals,^2–4^ semiconductors,^5,6^ superconductors,^7,8^ insulators,^9^ and even Mott insulators.^10,11^ In the case of halide Mott insulators, such as CrI_3_,^10^ CrBr_3_, CrCl_3_, VCl_3_ or NiI_2_ magnets, the twist degree of freedom drives the system to different magnetic states depending on the angle. However, their Mott nature remains unaffected due to the large mismatch between the Mott energy scale (3–4 eV) and the interlayer energy scale (100–300 meV).^12–17^

A promising family of two-dimensional Mott insulators are 1T transition metal dichalcogenides (TMDs).^18–21^ These include 1T-TaS_2_, 1T-TaSe_2_, 1T-NbSe_2_, and 1T-NbS_2_, which feature a charge density wave distortion (CDW), leading to a “star-of-David” (SoD) unit cell with 13 Ta (Nb) atoms and a nearly flat band emerging from the distortion itself.^22^ The nearly flat band inherits the correlations from the Ta (Nb) atoms, leading to a Mott insulating state^18,19,23^ with a reduced Mott scale of around 200 meV due to the CDW nature of the flat band.^24^ In the monolayer limit, this gives rise to a Mott insulating state with geometrically frustrated magnetism, becoming a potential platform for quantum spin liquid states.^19,23^ In contrast, the coupling between layers in bulk 1T-TaS_2_ overcomes the Mott gap and yields an insulating state that is driven by interlayer coupling rather than Mott correlations.^25^ While the physics of both monolayer and bulk have been studied in detail, potential emergent phases in twisted 1T-TaS_2_ and its related 1T family of dichalcogenides have remained largely unexplored. Twisted 1T-TaS_2_ bilayers have been synthesized, and their structure has been studied.^26^ *Ab initio* methods have predicted a metal–semiconductor transition and an insulating gap opening, respectively, in two relaxed structures of 1T-TaS_2_ bilayers for large twist angles,^27^ but only recently have the moiré modulations of the electronic structure of 1T-TaSe_2_ been probed with scanning tunneling microscopy (STM).^28^ Twisted TMDs, including others such as CDW 1T-NbSe_2_,^29^ 2H-TaS_2_ ^30,31^ and twisted TMD heterostructures,^32–35^ provide a variety of emergent phenomena achieved in recent years through twist engineering.

Here, we present a model of twisted 1T-TaS_2_ bilayers and analyze their electronic structure. We show that, due to the spatially dependent coupling between layers, the moiré pattern directly imprints a profile of many-body and single-particle gaps in real space. In particular, the magnetization profiles obtained by the model reveal that certain regions of the moiré develop local magnetic moments stemming from correlations, whereas others remain nonmagnetic due to an interlayer-driven single-particle gap. We also show the moiré modulated spectral signatures emerging from the coexisting many-body and single-particle correlated states. Finally, we demonstrate that an interlayer bias can be used to induce spatially modulated charge transfer, leading to a controllable local Mottness of the mosaic. Our results establish a new van der Waals platform to engineer emergent electronic states by controlling the degree of correlations through a moiré pattern.

1T tantalum dichalcogenides 1T-TaS_2_ and 1T-TaSe_2_ have a layered triangular lattice structure and present low-temperature commensurate CDW (CCDW) phases in which groups of 13 Ta atoms are clustered to form a 6-pointed star unit cell (SoD), each hosting one conduction electron in a $d_{z^{2}}$ orbital localized on the central Tantalum atom.^36–38^ A $\sqrt{13}\times \sqrt{13}$ SoD superlattice emerges, as depicted in Figure 1(a). The electronic structure of a monolayer in the CCDW phase can be described with a Wannier Hamiltonian for the nearly flat bands in a triangular lattice in which each site represents the Wannier state of a SoD.^25,39–41^ We can then build a half-filled Hubbard model Hamiltonian of this triangular lattice *H*_mono_ = *H*_intra_ + *H*_U_ to characterize the monolayer Mott insulating phase.^25^ *H*_intra_ is the tight-binding term that considers up to third-nearest-neighbor interactions, and the local interaction term *H*_U_ is the projected onsite electronic repulsion in the Wannier orbitals.

1 $$H_{\mathrm{intra}}=\underset{i,j,s}{\sum }t_{ij}c_{i,s}^{†}c_{j,s}+\mathrm{h.c.}$$

2 $$H_{U}=U\underset{n}{\sum }c_{n,↑}^{†}c_{n,↑}c_{n,↓}^{†}c_{n,↓}$$

where *t*_*ij*_ denotes the hopping between sites *i* and *j* and $c_{i,s}^{†}$ and *c*_*i*,*s*_ are the creation and annihilation operators of an electron with spin polarization *s* = ↑, ↓ at site *i*.

**Figure 1:**
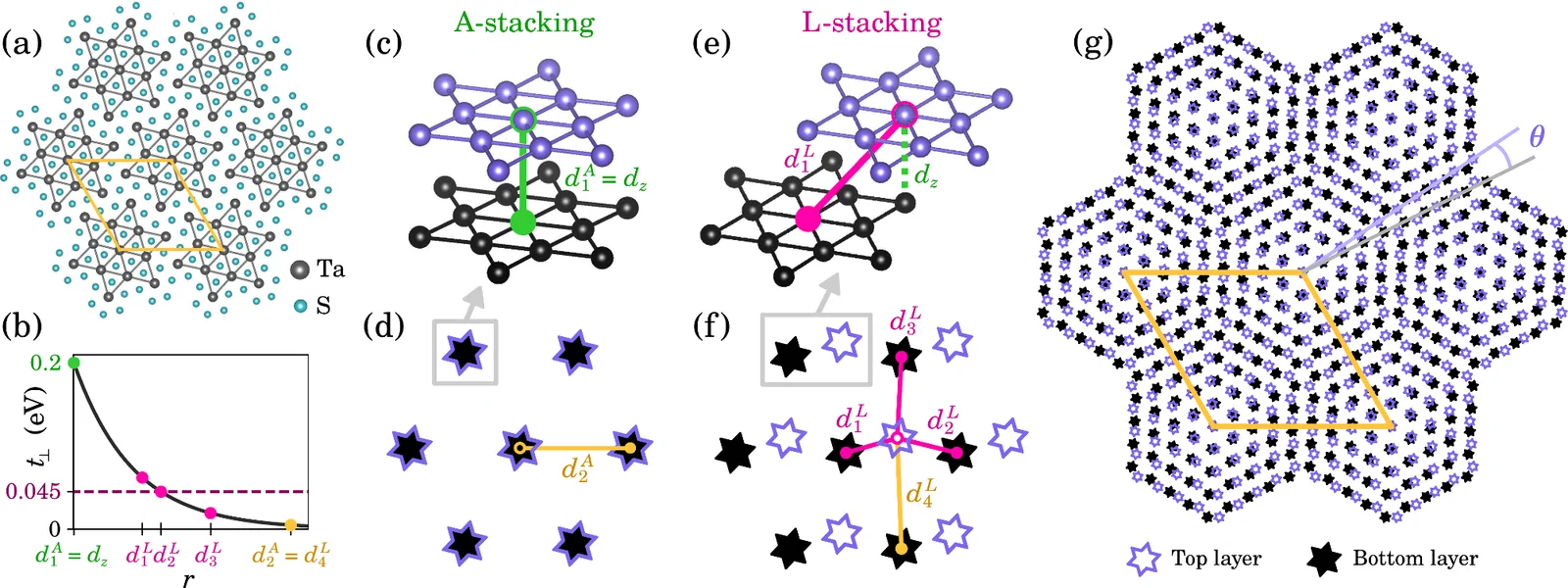
(a) CCDW atomic structure of monolayer 1T-TaS_2_ (top view), depicting the closest bonds between Ta atoms. CCDW supercell delineated in yellow. (b) Hopping *t*_⊥_ considered in the bilayer model between two SoD sites in different layers as a function of the distance between them *r* for τ = 0.2 eV.^25^ (c–f) Stacking orders found in bulk 1T-TaS_2_. (c, e) Ta atoms of the 2 SoD structures that make up the unit cells of A-stacking (c) and L-stacking (e). (d, f) Top view of the SoD sites of the A-stacked (d) and L-stacked (f) bilayers. Star symbols represent the center of each SoD structure, where the electrons are localized. (g) Moiré structure of twisted bilayer 1T-TaS_2_ with twist angle θ = 7.34° (top view).

The nearest-neighbor intralayer hopping parameter of 1T-TaS_2_ is taken as *t*_1_ = 0.05*U*,^42^ and the second- and third-nearest-neighbor hoppings preserve the ratios *t*_2_ = −0.23 *t*_1_ and *t*_3_ = −0.27 *t*_1_ stemming from the density functional theory electronic dispersion.^41^

The interaction term *H*_U_ can be solved at the mean-field level $H_{U}\approx H_{U}^{MF}$ by performing a noncollinear mean-field decoupling of the form

$$c_{n,↑}^{†}c_{n,↑}c_{n,↓}^{†}c_{n,↓}\approx \left\langle c_{n,↑}^{†}c_{n,↑}\right\rangle c_{n,↓}^{†}c_{n,↓}+\left\langle c_{n,↓}^{†}c_{n,↓}\right\rangle c_{n,↑}^{†}c_{n,↑}-\left\langle c_{n,↓}^{†}c_{n,↑}\right\rangle c_{n,↑}^{†}c_{n,↓}-\left\langle c_{n,↑}^{†}c_{n,↓}\right\rangle c_{n,↓}^{†}c_{n,↑}$$

The previous replacement gives rise to a mean-field ansatz that is explicitly rotationally symmetric and enables the emergence of general noncoplanar magnetic textures. The mean-field Hamiltonian takes the form

3 $$H_{U}^{\mathrm{MF}}=\underset{n,\alpha ,s,s^{\prime }}{\sum }J_{n}^{\alpha }\cdot \sigma_{s,s^{\prime }}^{\alpha }c_{n,s}^{†}c_{n,s^{\prime }}$$

where σ^α^ is the spin Pauli matrix and $J_{n}^{\alpha }$ is the self-consistent interaction-induced exchange field given by $J_{n}^{\alpha }=-U/2\underset{s,s^{\prime }}{\sum }\sigma_{s,s^{\prime }}^{\alpha }\left\langle c_{n,s}^{†}c_{n,s^{\prime }}\right\rangle$ . The interacting problem is solved by using a variational Hartree–Fock wave function $\left|\Omega_{\mathrm{MF}}\right\rangle =\underset{n}{\prod }\psi_{n}^{†}\left|0\right\rangle$ , where |0⟩ is the empty state and $\psi_{n}^{†}$ is the variational single-particle state, which is computed by solving the mean-field equations self-consistently.^43^

The lowest energy stacking structure of bulk 1T-TaS_2_ (AL-stacking) exhibits the two alternating stacking arrangements displayed in Figure 1(c–f), labeled A-stacking and L-stacking in reference to the center Ta atom of the SoD (A) and its upper-right star point (L).^44^ Our model will capture not only both of these stacking arrangements but also any translation and twist angle between layers by taking an interlayer hopping of the form

4 $$H_{\mathrm{inter}}=\underset{{\left\langle ij\right\rangle }_{⊥},s}{\sum }t_{⊥}\left(r_{ij}\right)c_{i,s}^{†}c_{j,s}+\mathrm{h.c.}$$

where ⟨*ij*⟩_⊥_ denotes a pair of sites belonging to different layers. The interlayer hopping is taken as a function that decays exponentially with the distance between two SoD sites, as shown in Figure 1(b).

5 $$t_{⊥}\left(r\right)=\tau e^{\lambda \left(d_{z}-r\right)}$$

*d*_*z*_ = 5.9 Å^45^ is the minimal distance between two layers of 1T-TaS_2_. The decay parameter λ = 0.49 Å^–1^ is chosen to reproduce bulk 1T-TaS_2_ parameters fitted to STM measurements,^25^ specifically those of the A-stacked nearest-neighbor distance $d_{1}^{A}=d_{z}(t_{⊥}\left(d_{1}^{A}\right)=0.2\;\mathrm{eV})$ (ref 25) and the L-stacked second-nearest-neighbor distance $d_{2}^{L}(t_{⊥}\left(d_{1,2,3}^{L}\right)=0.045\;\mathrm{eV})$ (ref 25). The value of the hopping strength factor τ ≡ *t*_⊥_(*d*_*z*_) that would correspond to the aforementioned model is τ = 0.5*U* (as *U* = 0.4 eV^25^). It is worth noting that τ can be experimentally increased with external uniaxial pressure, so in the following, we will explore the effect of this factor, while examining in greater detail a representative case in the realistic regime (τ = 0.7*U*). The distance-dependent interlayer hopping term allows the model to capture generic displaced stackings, as well as twisted layer stackings in which moiré periodicity emerges, as illustrated in Figure 1(g). It should also be noted that the interlayer distance *d*_*z*_ in twisted 1T-TaS_2_ bilayers is known to be somewhat sensitive to the twist angle^26,27^ due to atomic relaxations. An overall shift could be taken into account by the model simply by changing the interlayer hopping function, having a similar effect to a change in τ. A variation of the relaxed distances across the moiré would be another possibility that could moderately influence the mosaic phases presented.

The full Hamiltonian for a generic twisted bilayer finally becomes

6 $$H=H_{\mathrm{intra}}+H_{\mathrm{inter}}+H_{U}^{\mathrm{MF}}$$

It is worth noting that the previous mean-field ansatz leads to a symmetry-broken magnetic state in the Mott regime. As a result, potential quantum spin liquid states^23,46^ are not captured with the previous formalism nor does it capture finite-temperature effects, leading to a paramagnetic Mott state. The mean-field ansatz nevertheless will capture the competition between Mott physics and the hybridization gap even in those regimes, as such phenomenology stems from the competition between kinetic energy and interactions.

We first analyze the phase diagram for the aligned bilayers as given by the model above, which can be used as a reference for calculations of twisted bilayers. Figure 2(a) shows that the mean-field calculations using the described model predict that, when increasing the interlayer hopping strength τ, the magnetization of the sites in the A-stacked bilayer quickly decreases, becoming completely nonmagnetic for τ ≥ 0.5*U*. In contrast, the magnetization of the L-stacked bilayer sites only shows a slight decrease from the monolayer case, τ = 0 (noninteracting layers), which is known to be a Mott insulator. The results in the regime of sizable coupling between layers, τ ≥ 0.5*U*, are consistent with previous predictions,^25^ which show that a bulk sample of TaS_2_ (A-L alternating stacking) terminating in an A-stacked bilayer opens a hybridization gap and is band insulating, while one with an L-stacked bilayer termination hosts surface Mott insulating states. In the following analysis, we consider the representative case τ = 0.7*U* in order to focus on the realistic regime.

**Figure 2:**
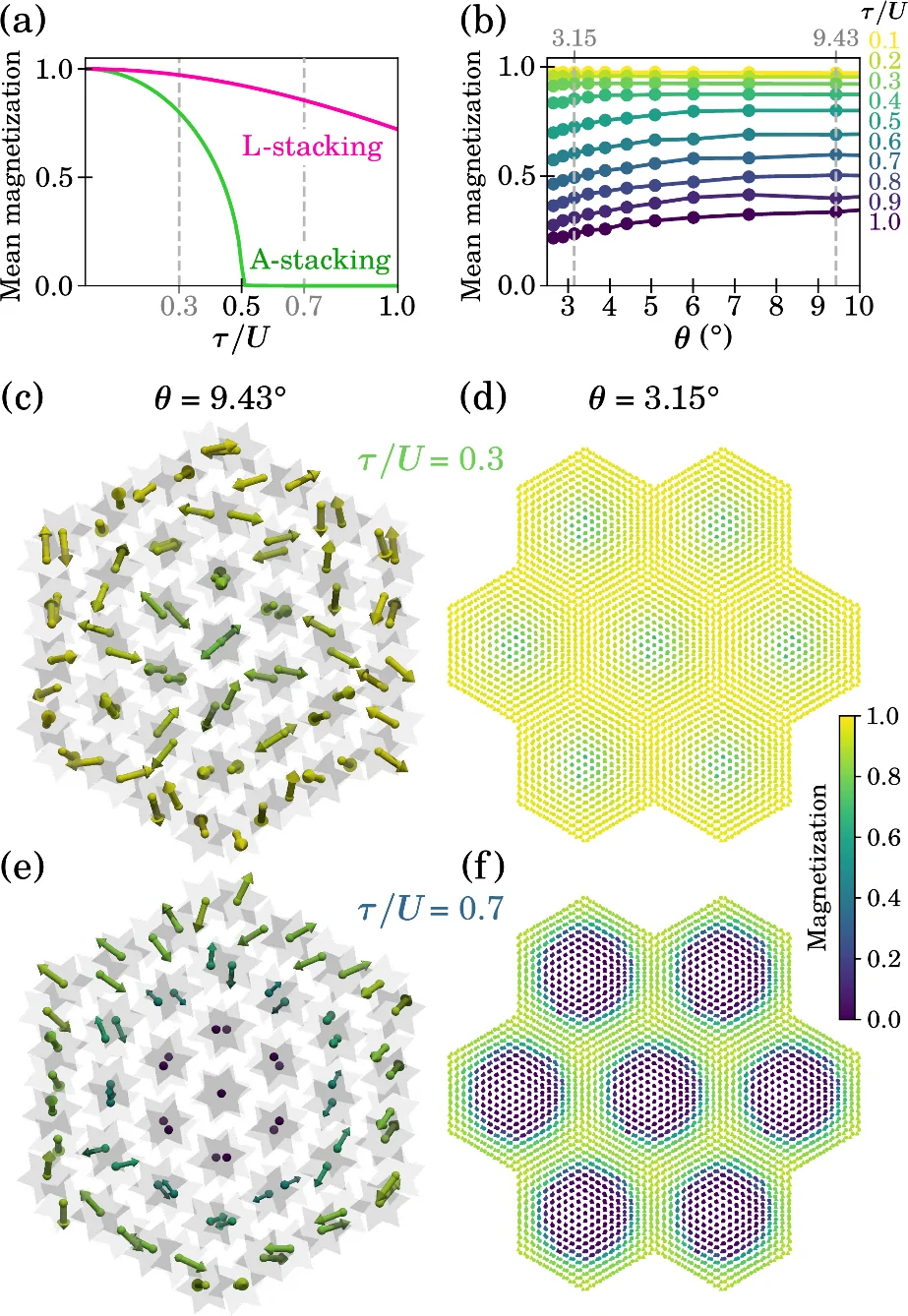
(a) Average absolute magnetization of each SoD site for the A-stacked and L-stacked 1T-TaS_2_ bilayers. Dashed lines highlight the values of τ of the twisted calculations shown in (c–f). (b) Average absolute magnetization of each SoD site for 1T-TaS_2_ bilayers twisted at different angles θ and considering different interlayer hopping strengths τ. Dashed lines highlight the angles θ shown in (c–f). (c–f) Top view of the magnetization on each SoD site in 1T-TaS_2_ bilayers with twist angles θ = 9.43° (c, e) and θ = 3.15° (d, f) in the low interlayer coupling regime, τ = 0.3*U* (c–d), and the high coupling regime, τ = 0.7*U* (e–f). (c, e) Magnetization vectors on each SoD site of a moiré supercell. (d, f) Norm of the magnetization of each site for multiple moiré supercells.

We now address moiré bilayers at different twist angles. Adding a twist angle between the two layers introduces local variations of interlayer neighbor distances, which in turn modulate the local interlayer hopping. We observe (Figure 2) that sites with closer interlayer neighbors (A-like regions) have reduced magnetization compared with sites whose interlayer neighbors are farther away (L-like regions). The effect is particularly strong in the cases where τ > 0.5*U*, as seen in Figure 2(e–f) for τ = 0.7*U* with twist angles θ = 9.43° and θ = 3.15°, respectively. In these cases, nonmagnetic regions appear in the center of the moiré supercells, where the interlayer coupling between SoD sites is similar to that of the A-stacking order. Even for a low interlayer hopping strength τ = 0.3*U* in Figure 2(c–d), the A–L contrast remains noticeable. This is analogous to the difference in the nontwisted results for these τ values (see dashed lines in Figure 2(a) for comparison). Figure 2(b) shows the average absolute local magnetization for different commensurate moiré twist angles and interlayer hopping strength values. As expected from the nontwisted results in Figure 2(a), Figure 2(b) demonstrates the effect of higher hopping strength between the layers decreasing the mean magnetization. Along with it, we observe that decreasing the twist angle has a smaller effect in lowering the mean magnetization.

It is worth noting that the local quenching of the Mott state can enable the promotion of further exotic magnetic states beyond the symmetry-broken solutions considered in our calculations. In particular, superlattice effects alone are potentially able to drive a magnetically ordered state into a quantum spin liquid state. While a purely triangular Heisenberg model shows a magnetically ordered state, a 2 × 2 triangular superlattice with a quenched site realizes a Kagome lattice, which shows a quantum spin liquid regime.^46^ A further paradigmatic example is the maple-leaf lattice,^47–49^ which can be understood as a locally quenched antiferromagnetic triangular superlattice. Recent results show that such a model sustains a variety of frustrated states, including spin liquids, enabled by the enlarged superlattice structure. These suggest that twisted bilayers can provide a potential platform to realize quantum magnetic states even when the monolayer develops magnetic order. Such spin liquid states, however, would require a methodology beyond the one presented in our manuscript, requiring either tensor network methods or approximate auxiliary Fermion calculations.

Another point to note is that the inclusion of spin–orbit coupling in the model could affect the emergent magnetic phase in the moiré system and renormalize the gap. However, the Mott mosaic phenomenology obtained by this model is not qualitatively affected by the inclusion of spin–orbit coupling, as the interplay between interlayer hopping and onsite interactions is the driving mechanism for the Mott mosaic phases. Strong anisotropy could drive the ground state to a more collinear AFM between layers^12,14,17^ as compared to the noncollinear spin texture obtained through this mean-field approach. Alternatively, the combination of spin–orbit coupling and noncollinear magnetism could also induce emergent ferroelectric polarization through the inverse Dzyaloshinskii–Moriya interaction.^12,13,15^

We now analyze the spectroscopic signatures, which are directly accessible with STM.^23,28^ We first show how the model accounts for the spectroscopy of nontwisted bilayer stackings. Figure 3(a) shows the band gap dependence as a function of interlayer hopping strength for both A- and L-stackings, indicating that at τ = 0.5*U* the hybridization gap of the A-stacked bilayer starts growing linearly with τ. This demonstrates the band insulating character of the A-stacked bilayer in the τ > 0.5*U* regime, while the L-stacked bilayer remains a Mott insulator (see Supporting Information for an extension of the model to phases beyond τ > *U*).

**Figure 3:**
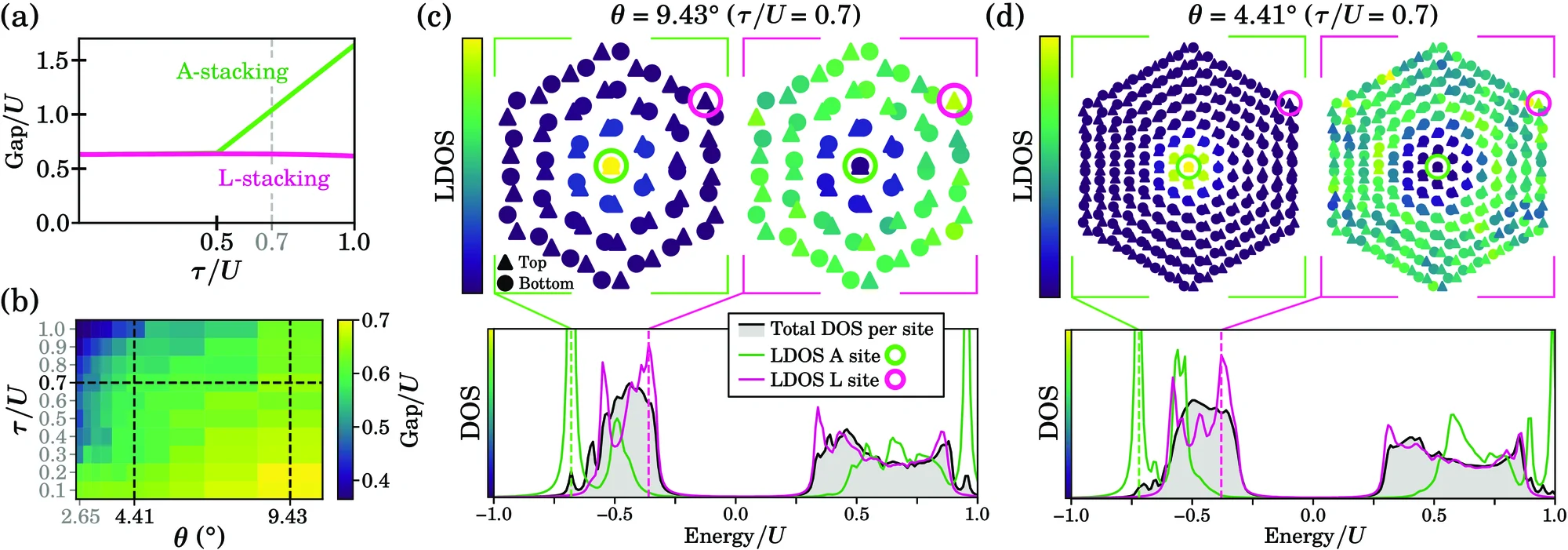
(a) Band gap of the nontwisted A-stacked and L-stacked 1T-TaS_2_ bilayers for different interlayer hopping strengths τ. The dashed line indicates the value of τ used in plots (c–d). (b) Band gap of twisted bilayers with different twist angles θ and interlayer hopping strengths τ. The value of τ and the twist angles used in plots (c–d) are highlighted. (c–d) Density of states of the 1T-TaS_2_ bilayer with interlayer hopping strength τ = 0.7*U* and twist angles θ = 9.43° (c) and θ = 4.41° (d). Bottom: Total DOS divided by the number of SoD sites in the moiré supercell alongside LDOS of sites A and L. Top: LDOS of every SoD site plotted as a top view in real space at peaks of the LDOS spectrum of the A site (left) and L site (right). Sites labeled “A” and “L” are circled.

We then calculate the local density of states (LDOS) of the twisted bilayer structures in the realistic τ regime and compare sites with a close interlayer neighbor, labeled “A”, against sites with farther neighbors, labeled “L”. Figure 3(b) shows the band gap for 1T-TaS_2_ twisted bilayer systems and its dependence on τ. The LDOS results indicate that the smallest gap corresponds to the L sites, and so, it is expected that the gap decreases with higher τ values. Additionally, we observe an increase in this Mott gap for larger twist angles. As seen in the lower panels of Figure 3(c–d) for two different twist angles, the gap of the A-site LDOS is significantly larger than the L-site LDOS for any twist angle, consistent with hybridization in the central region of the moiré supercells where the sites are nonmagnetic.

Furthermore, the difference in the LDOS gaps produces modulations of the LDOS in real space, as shown in the top real-space plots of Figure 3(c–d). Two examples of this moiré modulation are illustrated for each twist angle, one corresponding to a peak in the A-site LDOS (green) and another to a peak in the L-site LDOS (magenta). This spatial LDOS modulation in twisted bilayer TaS_2_ could be experimentally resolved using STM, and in fact for twisted bilayer 1T-TaSe_2_, with a similar CDW structure, spatial modulation of the gaps has been found with this technique,^28^ although in this case the Mott insulating state is suppressed and becomes metallic. An extension of the model for higher interlayer coupling is included in the Supporting Information in order to better capture the behavior of 1T-TaSe_2_.

The low dimensionality of twisted van der Waals bilayers enables a high degree of tunability through external electric gates.^8,50–58^ Therefore, having characterized the emergent moiré Mott mosaic in twisted 1T-TaS_2_ bilayers, we show how the correlated state can be externally controllable with an external interlayer gate bias. A bias between layers gives rise to an additional term in the Hamiltonian of the form

7 $$H_{V}=V\underset{n,s}{\sum }\chi_{n}c_{n,s}^{†}c_{n,s}$$

where χ_*n*_ = ±1 for the upper and lower layers, respectively, and *V* controls the strength of the interlayer bias (2*V*), as represented in Figure 4(a). In particular, such a term will drive the monolayers away from half filling, promoting charge transfer from one layer to the other.^21,59,60^ As the bias term *V* increases, charge transfer occurs at different rates in the A- and L-stacked bilayers, as shown in Figure 4(b). At low interlayer bias, the Mott insulating L-stacked -bilayer is more resistant to charge transfer. However, at *V* ≈ 0.5*U*, charge transfer occurs at an increased rate, and so for larger bias, the difference in occupation between layers becomes larger for L-stacking. This pattern also arises in the twisted bilayers, as shown in Figure 4(d–e) when comparing the occupation of the A sites against the L sites. Figure 4(h–i) displays the occupation of every site of the moiré supercells in real space, revealing the resulting bias-dependent modulation at the moiré scale. We further analyze the magnetization profiles with increasing *V*. Figure 4(c) shows that the A-stacked bilayer remains nonmagnetic as expected, whereas the magnetization in the L-stacking sites decreases at a rate similar to the change in their occupation. This behavior is found locally in the A and L sites of the twisted layers, as shown in Figure 4(f–g), and Figure 4(j–k) illustrates this decreasing magnetization of the antiferromagnetic Mott regions as *V* increases.

**Figure 4:**
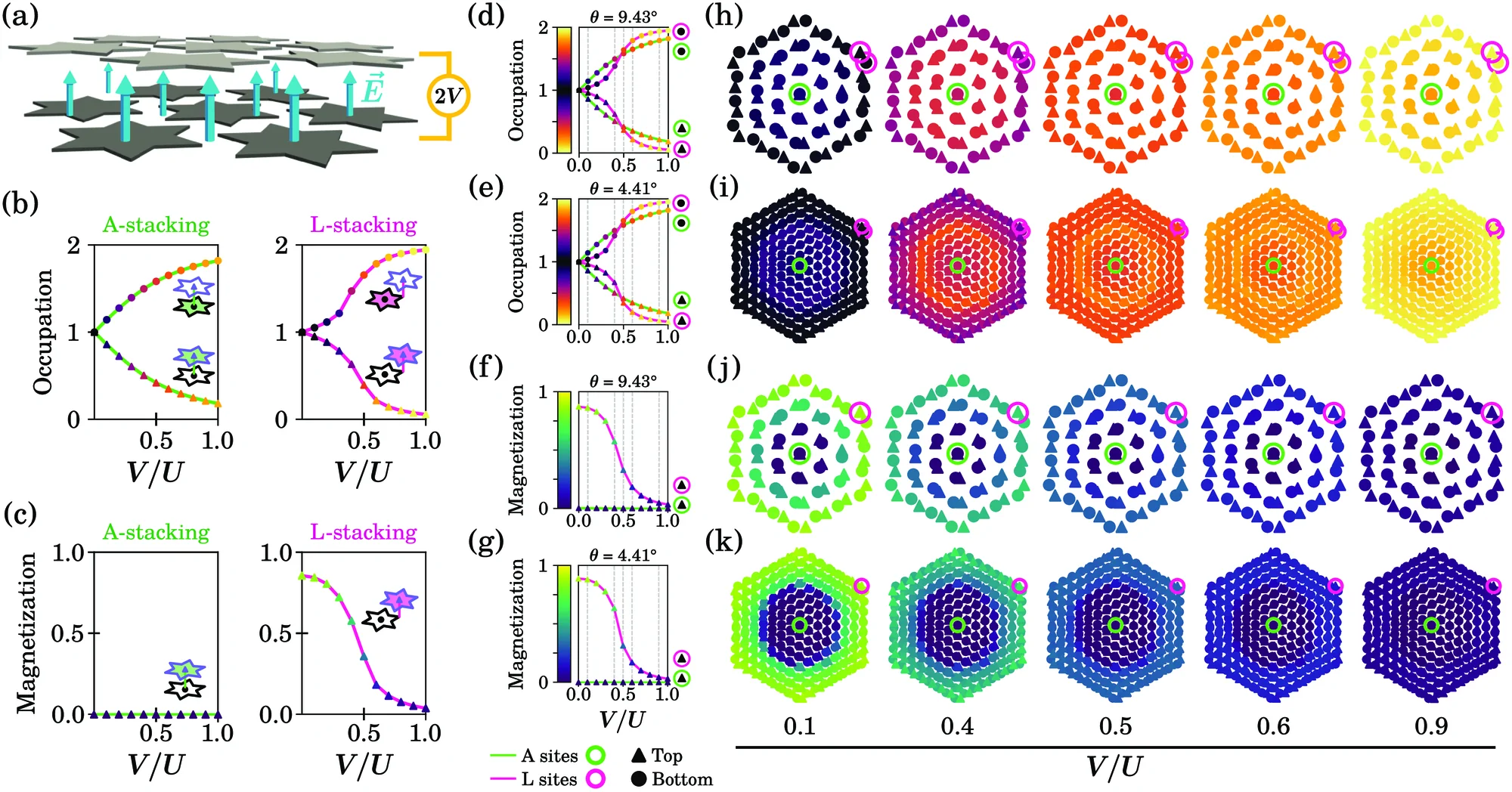
(a) Schematic of a bias voltage 2*V* applied between two twisted 1T-TaS_2_ layers. (b) Occupation of the top and bottom sites of A-stacking in contrast with those of L-stacking with increasing *V* for interlayer hopping strength τ = 0.7*U*. (c) Magnetization of the top sites of A- and L-stacking with increasing interlayer bias *V* for τ = 0.7*U*. (d–e, h–i) Occupation of the A and L sites (d–e) and of each site of the moiré supercell (h–i) of bilayer structures with twist angles θ = 9.43° (d, h) and θ = 4.41° (e, i) with increasing *V* for τ = 0.7*U*. (f–g, j–k) Magnetization of the top A and L sites (f–g) and of each site of the moiré supercell (j–k) of bilayer structures with twist angles θ = 9.43° (f, j) and θ = 4.41° (g, k) with increasing *V* for τ = 0.7*U*. The corresponding L sites are circled (h–k). Note that the magnetization in the bottom sites, not shown in (c, f–g), is equal in magnitude with the opposite direction.

We now address the effect of interlayer bias on the density of states of the bilayer systems. In the nontwisted structures, a distinct behavior of the two stacking orders in the presence of *V* appears. Increasing *V* gradually increases the size of the gap in the A-stacked bilayer, as shown in Figure 5(a). In contrast, Figure 5(b) presents a more dramatic electrical control of the electronic structure of the L-stacked bilayer, as the gap initially decreases, closing near *V* ≈ 0.5*U*, and then increases for higher bias. The LDOS of the A and L sites of the twisted bilayers, located in the center and edge of the moiré supercell as depicted in Figure 5(c–d), locally reflect the features observed in the A- and L-stacking orders: LDOS gap increasing in the A sites but closing and reopening in the L sites.

**Figure 5:**
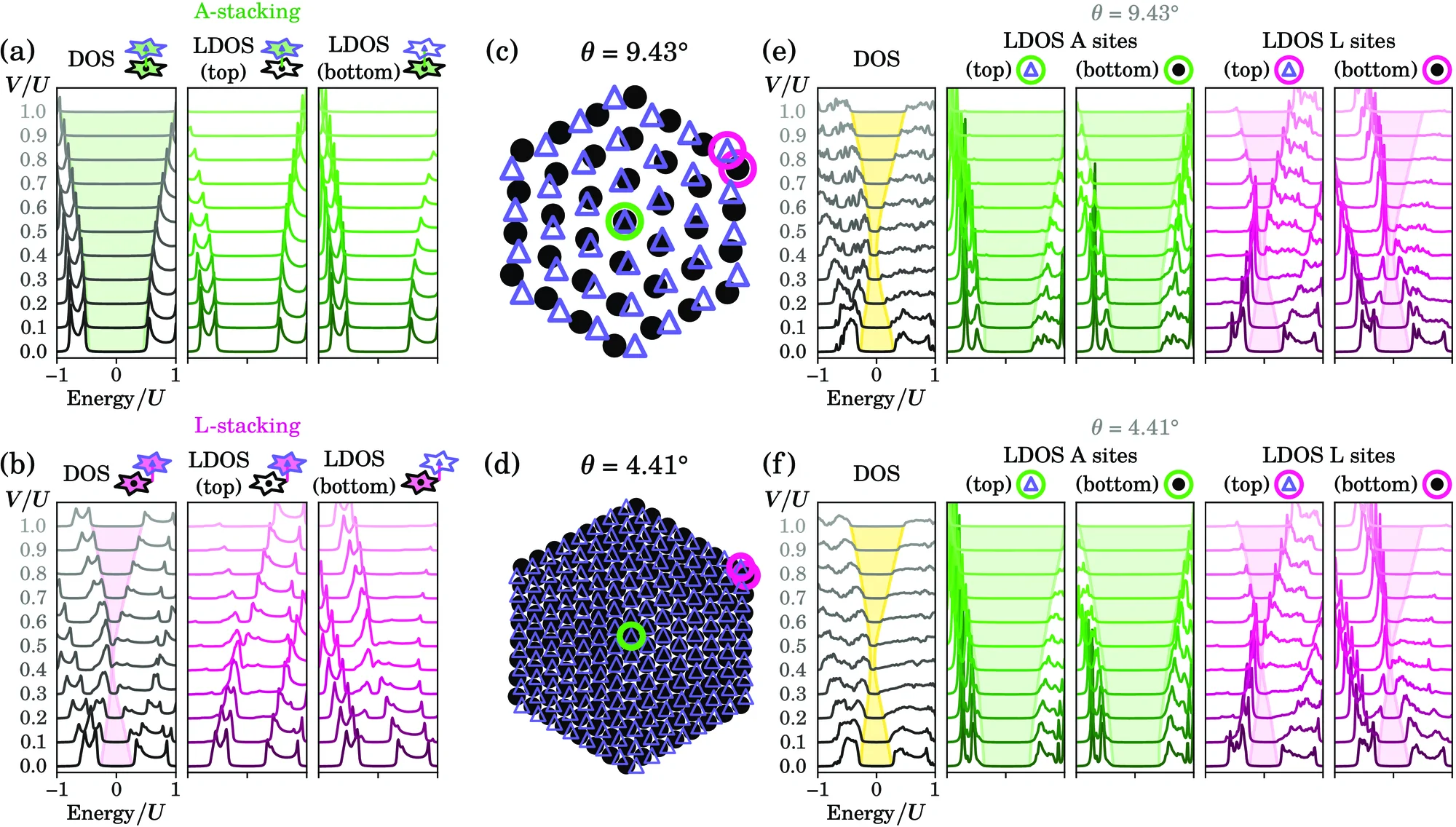
(a–b) DOS of the A-stacked (a) and L-stacked (b) 1T-TaS_2_ bilayers with interlayer hopping strength τ = 0.7*U* and LDOS of their top and bottom sites for increasing interlayer bias 2*V*. (c–d) Top view of bilayer lattice structures with twist angles θ = 9.43° (c) and θ = 4.41° (d) with A sites marked by a green circle and L sites marked by magenta circles. (e–f) For the 1T-TaS_2_ bilayers with τ = 0.7*U* and twist angle θ = 9.43° (e) and θ = 4.41° (f): total DOS, LDOS at the A sites in the top and bottom layers, and LDOS at the L sites in the top and bottom layers.

This dependence of the gap on an external bias provides an additional experimental signature of the Mott region of the moiré. Other possibilities to probe the Mott phases would include resolving the magnetic texture through spin-polarized STM techniques and using Inelastic Tunneling spectroscopy (IETS) to measure spin excitations (magnons or spinons) associated with the magnetic phases emerging in the Mott regions of the moiré system, as demonstrated in multiple experiments in 2D materials.^23,61–64^ Additionally, Kondo resonances at zero bias could provide solid proof of the Mott character of the gap.^65^ The spin response of the twisted bilayer system that could be probed with IETS is included in the Supporting Information.

The bias tunability of the Mott region (L sites), in contrast with the minimal changes in the A sites, may also indicate that changes in the electronic structure of the twisted bilayer system resulting from doping or superconducting states would be localized to this Mott region. These results establish the potential for electrically fine-tunable semiconducting devices based on the moiré Mott mosaic emerging in twisted 1T-TaS_2_ bilayers.

We have shown that the spatially dependent coupling in 1T-TaS_2_ leads to a competition between single-particle and correlation-driven Mott gaps. The moiré bilayer features a fully gapped state across the whole moiré, yet the origin of the gap is directly inherited from the local stacking of the moiré pattern. We have shown the emergence of a moiré mosaic with an arrangement of magnetic and nonmagnetic regions, corresponding to Mott and hybridized gaps, respectively. We demonstrated that the moiré Mott mosaic is tunable with an electric bias in a nonmonotonic way thanks to the coexistence of Mott and single-particle regions. Our results establish 1T-TaS_2_ twisted bilayers as promising candidates for tunable electronic and magnetic phases, providing a unique platform where Mott and single-particle gaps are directly imprinted through twist engineering.

## Supplementary Material



## Notes

Discussion of the generality of the model and extended calculations to lower intralayer and higher interlayer hopping as predicted for 1T-TaSe_2_; simulation of IETS signatures of the Mott mosaic (PDF)
