# Supplementary material for: Moiré Mott Correlated Mosaics in Twisted Bilayer 1T-TaS2
Source: Nano Lett. 2026 Aug 24;26(39):13177–84. doi: 10.1021/acs.nanolett.6c01799 (PMC13651619; doi:10.1021/acs.nanolett.6c01799)
Supplement: Supplementary file 1 [file nl6c01799_si_001.pdf]

Supporting Information for Moiré Mott correlated mosaics in twisted bilayer 1T-TaS_2

Ana Vera Montoto,[1] Jose L. Lado,[1] and Adolfo O. Fumega[1]

[1] *Department of Applied Physics, Aalto University, 02150 Espoo, Finland*

(Dated: August 17, 2026)

GENERALITY OF THE MODEL AND EXTENSION OF THE PARAMETER'S RANGE

Recent STM measurements on a twisted 1T-$TaSe_2$ layer on the bulk [1] find a spatial modulation of the gap corresponding to the moiré of the SoD superlattice. For this isostructural material to 1T-TaS_2, the hybridization of A-sites opens a gap, but the L-states host metallic states as opposed to the Mott insulating state our model predicts for 1T-TaS_2 bilayers. This difference motivates an extension of the parameter range of the proposed mean-field model to better capture the phenomenology in 1T-$TaSe_2$. Even though both materials host an insulating state in the monolayer, they display somewhat different phenomenology, mainly the coexistence of metallic and insulating surface states in bulk 1T-$TaSe_2$ while 1T-TaS_2 is insulating [2].

Robustness of the Mott mosaic for small intralayer hopping

Although more accurate results could be obtained by fitting the hopping parameters of the model to *ab initio* calculations of 1T-TaS_2 and $TaSe_2$, we can nevertheless probe the spatially varying phases in twisted bilayers in the regime of dominant U and interlayer hopping. Liu et al. [1], for instance, use for 1T-$TaSe_2$ lower intralayer hopping values to those of our model. The calculations in Fig. **S1** show the phases predicted by our model for the twisted bilayers with the intralayer hopping parameters that they considered for their model of 1T-$TaSe_2$ ($t_1 = 1.179$ meV $= 0.003\,U$, $t_2 = 0.214$ meV, and $t_3 = -1.461$ meV), and the same intralayer hopping strength $\tau = 0.7\,U$ as considered in Fig. 3–5. Fig. **S1**(a) shows a similar dependence of the average magnetization as for the original intralayer hoppings (see Fig. 2(b)). The moiré modulation of non-magnetic and antiferromagnetic sites is still reproduced as seen in Fig. **S1**(b) (see Fig. 2(f) for the original parameters), and so is the LDOS gap in Fig. **S1**(c,d) (see Fig. 3(c,d)).

These results demonstrate that dominant U and interlayer hopping over the intralayer hopping is enough to reproduce the spatial modulation of magnetization and local density of states that result from the Mott mosaic. This, however, is not enough to reproduce the 1T-$TaSe_2$ case, given that Mott states are present while in experiment these regions were metallic [1]. This is because the larger p orbitals of Se atoms as compared to S atoms are expected to increase the interlayer hopping, due to a larger overlap between the layers. As we will see in the next section, an increase in the interlayer hopping τ with respect to the onsite repulsion U can drive the system to a quasi-metallic state in some stacking regions.

1T-$TaSe_2$'s quasi-metallic–hybridization gap moiré in the higher τ/U regime

Liu et al. [1] predict with density functional theory a small gap in L-stacked 1T-$TaSe_2$ bilayers, which in STM measurements becomes metallic due to the weak coupling between the surface bilayer and the bulk sample. This small gap is consistent with the high interlayer coupling regime of our model. As demonstrated in Fig. **S2**(a), high τ diminishes the gap of the L-stacked bilayer and shows a transition from the Mott phase to a small-gap semi-metal. Both increasing τ and decreasing the decay parameter λ of the interlayer hopping dependence on distance, as shown in Fig. **S2**(b), increase the hopping to farther interlayer neighbors, resulting in the gap decreasing faster. In Fig. **S2**(c) we can see the transition to a phase with a small gap at high τ for twisted bilayers with different twist angles. Fig. **S2**(d) then shows the effect of both τ and λ on the gap of bilayers twisted with angle $\theta = 4.41°$. The decrease of the gap and the transition to semi-metallic states seems to be sharper and occur for significantly lower τ for twisted bilayers than it does in the L-stacked case. This difference might be due to the effect of the non-collinear magnetic order in the twisted case, although a more complete analysis of stacking configurations other than A and L could reveal a different τ dependence that is being reproduced in the twisted bilayers.

Fig. **S3** shows some examples of the LDOS modulation in twisted bilayers in the high τ case ($\tau = 1.5\,U$), comparing the smaller-gap spectrum of the L-sites inside the hybridization gap of the A-sites.

And completing the picture of these high interlayer hopping phases, Fig. **S4** shows the dependence of the magnetization with an extended range of τ. Both in the aligned stacking cases (Fig. **S4**(a)) and the twisted bilayers (Fig. **S4**(b–f)) we observe that the sites become non-magnetic when the small-gap phase is reached.

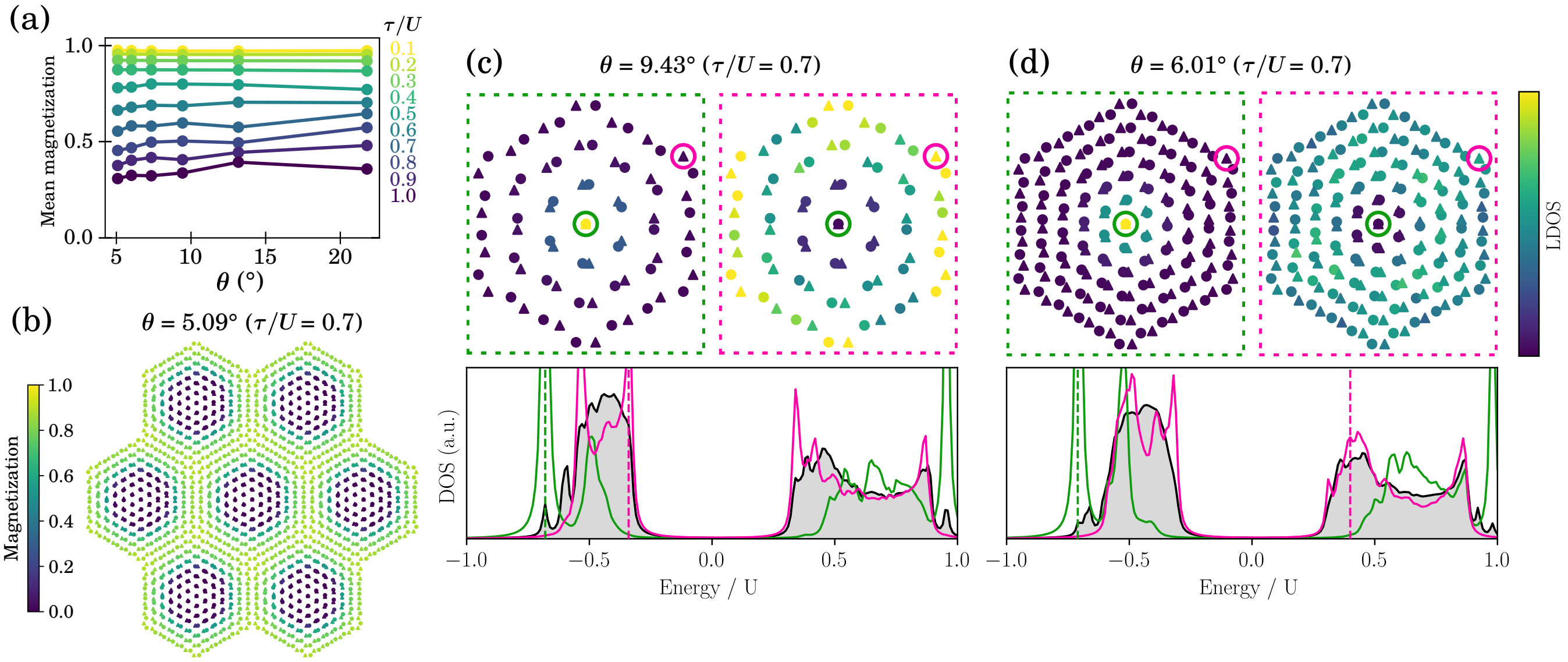

FIG. **S1**. Results for $t_1 = 1.179$ meV, $t_2 = 0.214$ meV, and $t_3 = -1.461$ meV, as taken in [1] for 1T-TaSe$_2$. **(a)** Average absolute magnetization of each SoD site for bilayers twisted at different angles θ and considering different interlayer hopping strengths τ. **(b)** Norm of the magnetization of each SoD in multiple moiré supercells of bilayers with twist angle $\theta = 5.09°$, for $\tau = 0.7\,U$. **(c,d)** Density of states of the bilayers with interlayer hopping strength $\tau = 0.7\,U$ and twist angles $\theta = 9.43°$ (c) and $\theta = 6.01°$ (d). Bottom: Total DOS divided by the number of SoD sites in the moiré supercell alongside LDOS of sites A and L. Top: LDOS of every SoD site plotted as a top view in real space, at peaks of the LDOS spectrum of the A site (left) and L site (right). Sites labeled "A" and "L" are circled.

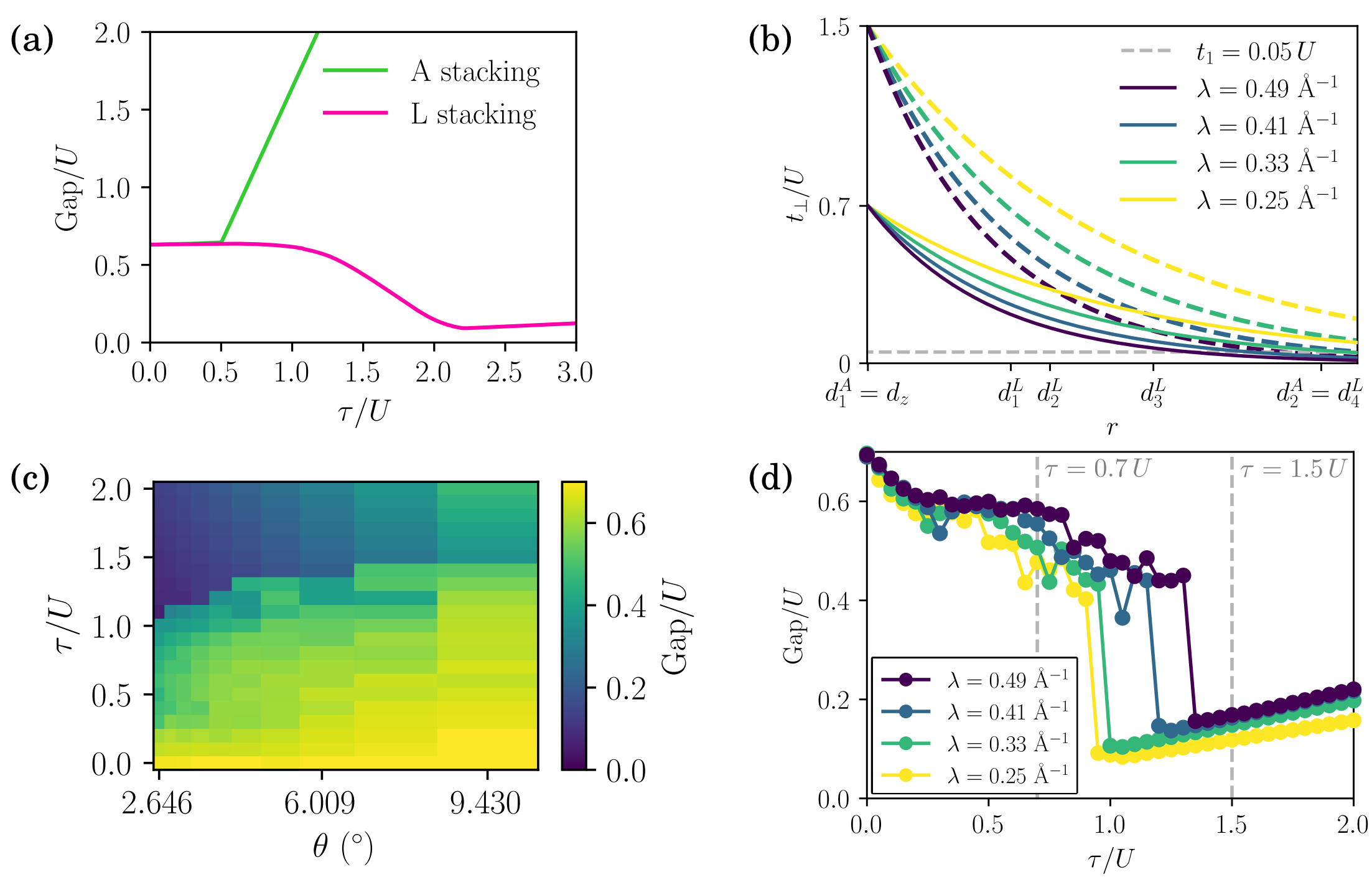

FIG. **S2**. **(a)** Band gap of the non-twisted A-stacked and L-stacked bilayers for the range of interlayer hopping strengths $\tau \in [0, 3\,U]$ (for $\lambda = 0.49$ Å^{-1}, extension of Fig. 3(a)). The dashed line indicates the value of τ used in Figs. 3–5. **(b)** Interlayer hopping function of distance r with different decay parameters λ for $\tau = 0.7\,U$ (continuous lines) and $\tau = 1.5\,U$ (dashed lines). The gray dashed line indicates the first neighbor hopping $t_1 = 0.05\,U$. **(c)** Gap dependence on τ predicted by the model for the twisted bilayer with twist angle $\theta = 4.41°$, considering different decay parameters λ. The dashed lines highlight $\tau = 0.7\,U$, used in Figs. 3–5, and $\tau = 1.5\,U$, used in Fig. S3.

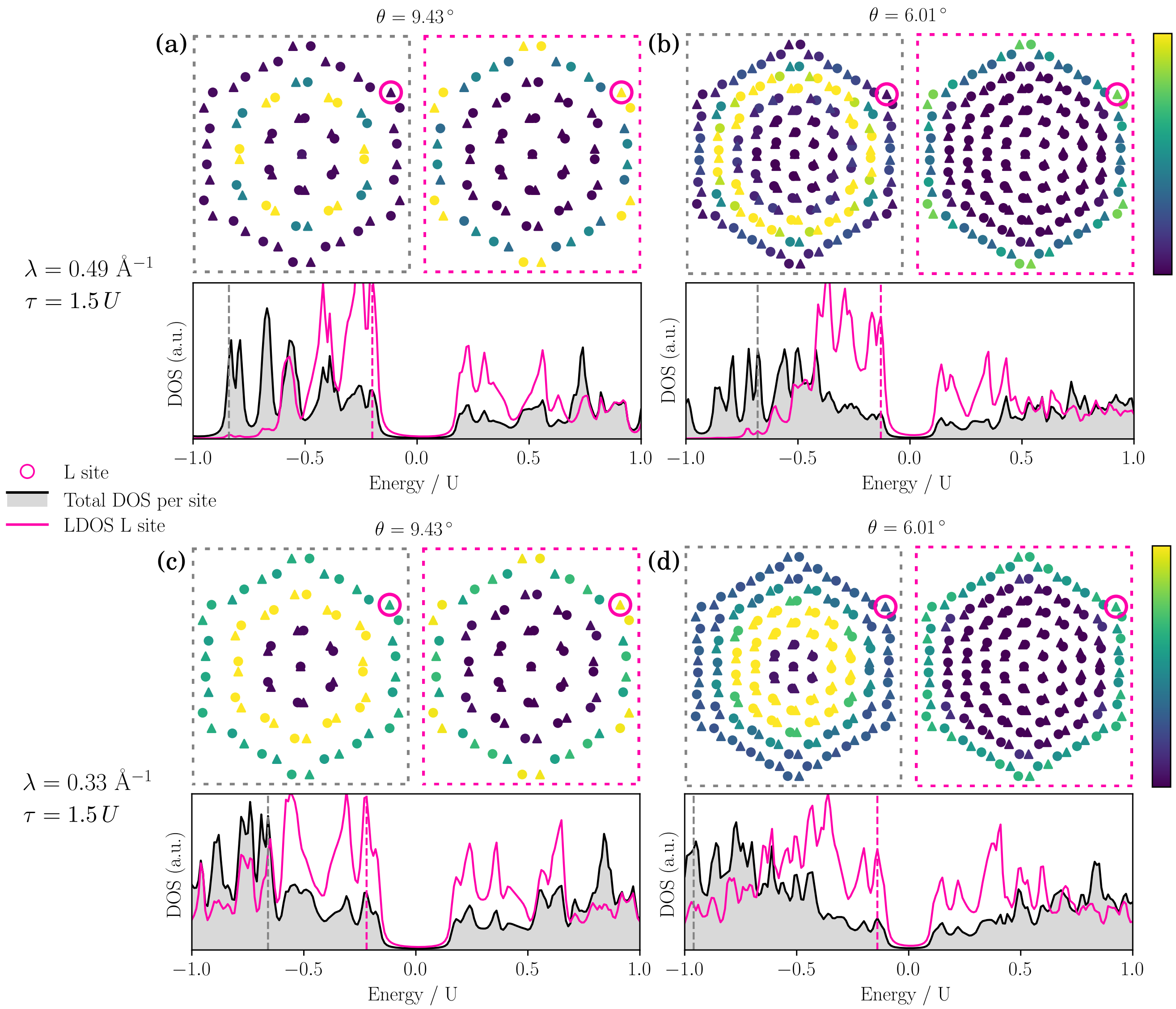

FIG. **S3**. Density of states of the twisted bilayers with interlayer hopping strength $\tau = 1.5\,U$, interlayer hopping decay $\lambda = 0.49$ Å^{-1} (a,b) and $\lambda = 0.33$ Å^{-1} (c,d), and twist angles $\theta = 9.43°$ (a,c) and $\theta = 6.01°$ (b,d). Bottom: Total DOS divided by the number of SoD sites in the moiré supercell alongside LDOS of site L. Top: LDOS of every SoD site plotted as a top view in real space, at the energies marked by the dashed gray line (left) and pink line (right). Sites labeled "L" are circled.

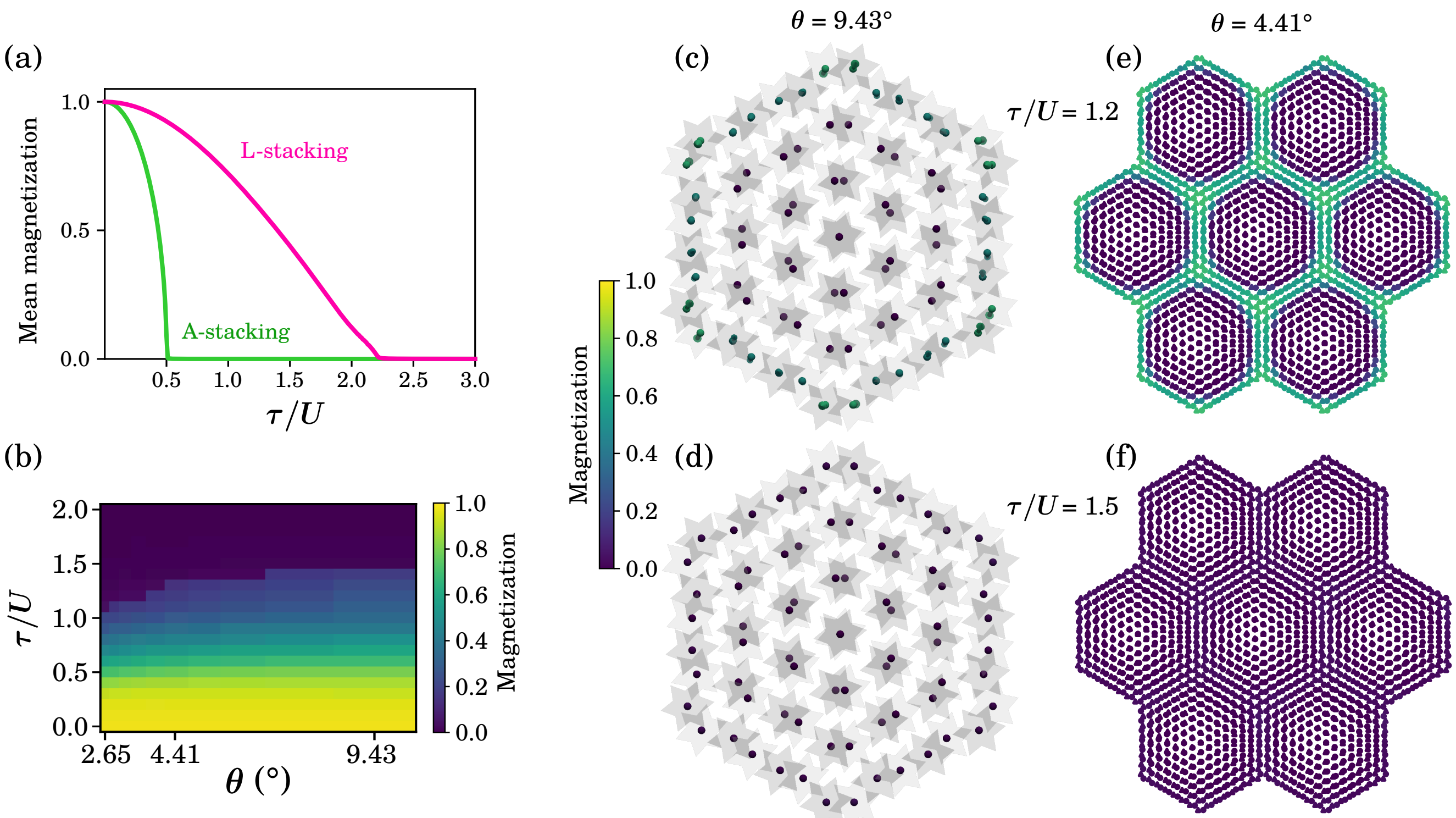

FIG. **S4**. **(a)** Average absolute magnetization of each SoD site for the A-stacked and L-stacked bilayers (extension of Fig. 2(a)). **(b)** Average absolute magnetization of each SoD site for bilayers twisted at different angles θ and considering different interlayer hopping strengths τ (extension of Fig. 2(b)). **(c–f)** Top view of the magnetization on each SoD site in bilayers with twist angles $\theta = 9.43°$ (c,d) and $\theta = 3.15°$ (e,f) for higher interlayer coupling $\tau = 1.2\,U$ (c,e), $\tau = 1.5\,U$ (d,f). (c,d) Magnetization vectors on each SoD site of a moiré supercell. (e,f) Norm of the magnetization of each site for multiple moiré supercells.

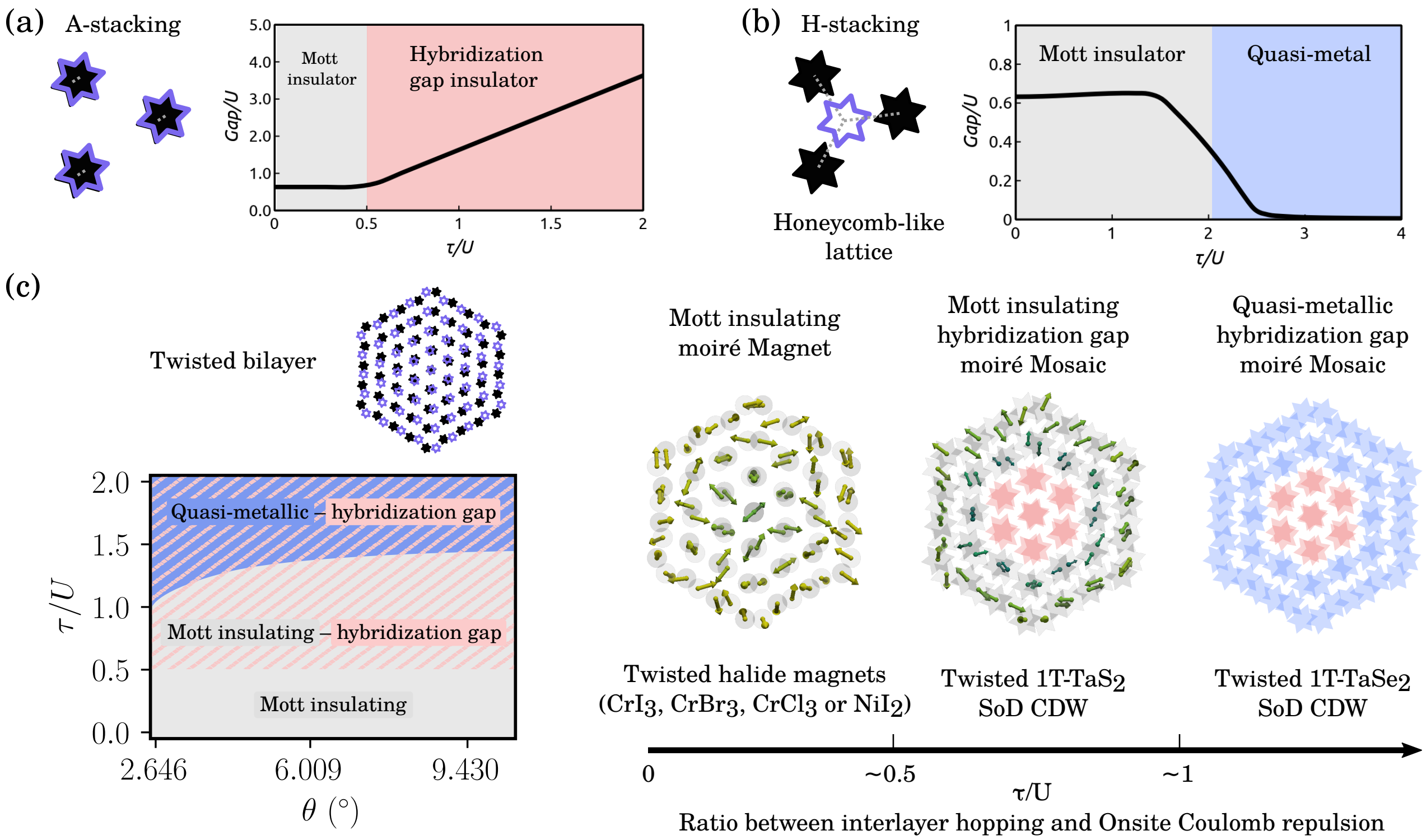

FIG. **S5**. **(a,b)** Band gap of the non-twisted A-stacked and H-stacked bilayers for the range of interlayer hopping strengths $\tau \in [0, 2\,U]$ $\tau \in [0, 4\,U]$ respectively (for $\lambda = 0.49$ Å^{-1}, used in the main text). The H-stacking corresponds to sites with three equally distant interlayer neighbors, displaying a honeycomb-like lattice. At large interlayer hopping τ, this mimics a graphene-like lattice, producing a semimetallic behavior. **(c)** Schematic of the different phases that can arise from the competition between interlayer hopping τ and onsite Coulomb repulsion U for twisted Mott bilayers. At strong U values, the system remains a Mott insulator at every stacking region. This phase corresponds to twisted halide magnets (CrI_3, $CrBr_3$, $CrCl_3$ or NiI_2 [3–8]). At strong τ values, the system develops a quasi-metallic state in the L and H regions and a strong hybridization-gap insulating area in the A stacking regions. This phase corresponds to twisted 1T-$TaSe_2$ SoD-CDW bilayers [1]. At similar values of τ and U, the twisted bilayer develops the moiré Mott mosaic phase analyzed in the main text for twisted 1T-TaS_2 SoD-CDW bilayers.

Generality of the model

To further demonstrate the generality of our model, and explain how it captures the quasi-metallic–insulating phase recently reported for twisted 1T-$TaSe_2$ bilayers [1], we present calculations of the gap (and its character based on the emergent magnetization) for the A-stacking and the H (honeycomb) stacking (Fig. S5(a) and S5(b)). The H-stacking corresponds to three equally distant interlayer neighbors (in contrast to the previously discussed L-stacking). This configuration effectively behaves as a honeycomb lattice. We can observe that at large interlayer hopping τ/U, the electronic structure mimics a graphene-like lattice, thus producing semimetallic behavior (Fig. S5(b)). This allows us to rationalize the quasi-metallic–insulating behavior reported in twisted 1T-$TaSe_2$ bilayers [1], where the strong τ/U value drives the system to a phase where quasi-metallic regions (L- and H- stackings) coexist with large hybridization gap areas (A-stacking). Apart from this, the model also captures the small interlayer hopping regime. In this scenario, the Mott behavior dominates in all the stacking regions, corresponding to the previously analyzed cases of twisted halide magnets CrI_3, $CrBr_3$, $CrCl_3$, or NiI_2 [3–8]. Finally, at intermediate τ/U values, the twisted system displays the Mott insulating hybridization gap phase analyzed in the main text, corresponding to twisted TaS_2 bilayers. All these emergent phases as a function of the τ/U ratio are diagrammatically summarized in Fig. S5(c).

DETECTION OF MAGNETISM FROM INELASTIC SPECTROSCOPY

From an experimental point of view, the Mott correlated state can be distinguished from the bonding-antibonding gap from its spin excitations. In particular, in dI/dV STM spectroscopy, the conductance has contribution both from elastic and inelastic tunneling. The elastic tunneling directly reflects the electronic density of states, and is non-zero only above the single-particle gap. The inelastic contribution can be non-zero below the electronic gap, and stems from many-body excitations in the host material. Specifically, the inelastic contribution in a Mott correlated system directly reflects the many-body magnetic excitations below the electronic gap, taking the form [9]

$$\frac{d^2 I}{dV^2}_{IETS} \sim A(\omega), \tag{1}$$

where $A(\omega)$ is the spatially resolved many-body spin structure factor, that takes the form [9]

$$A(\mathbf{r}_\alpha, \omega) = \sum_\sigma \langle \Omega | S^\sigma_\alpha \delta(\omega - H + E_{GS}) S^\sigma_\alpha | \Omega \rangle, \tag{2}$$

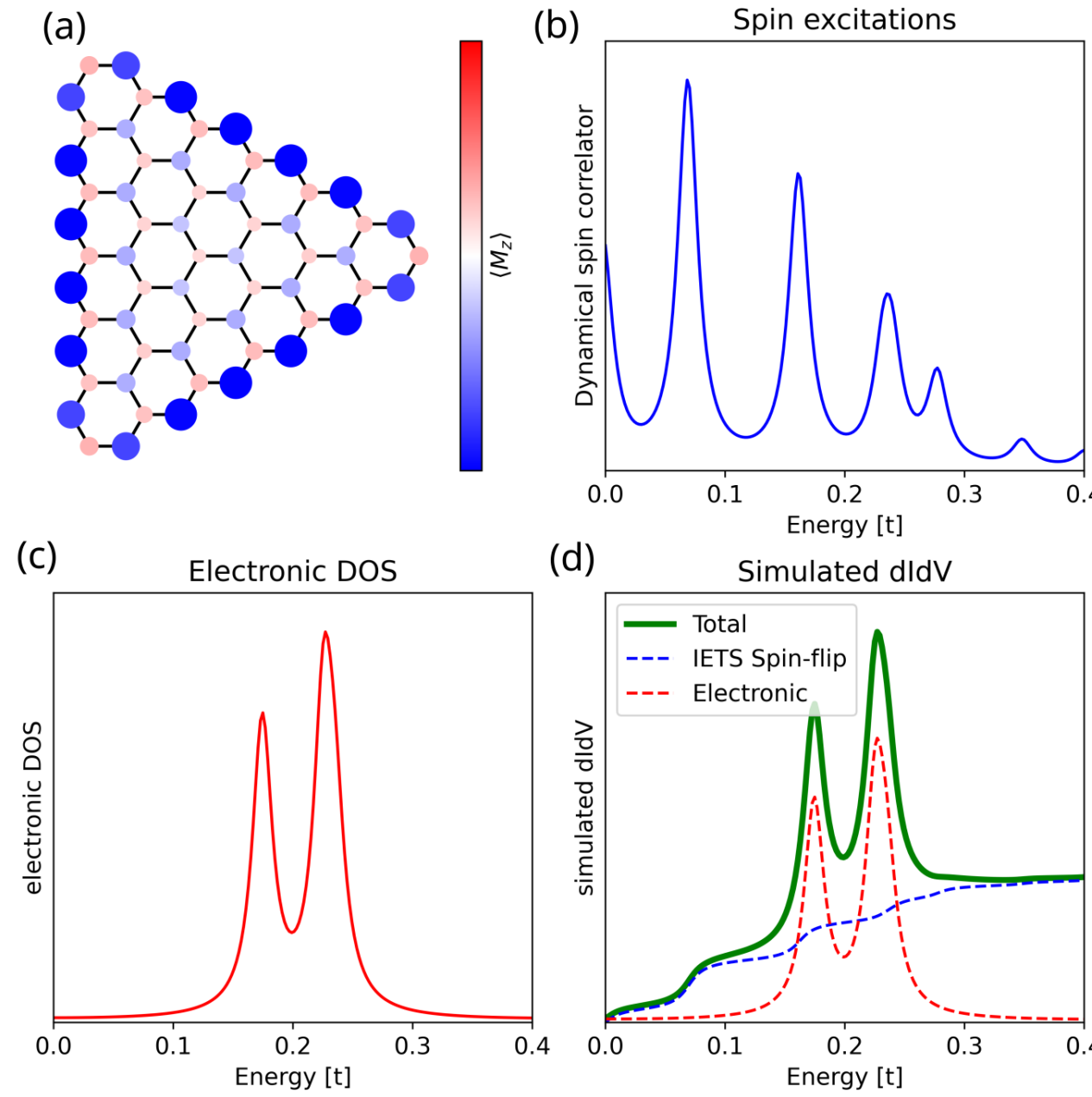

FIG. **S6**. Self-consistent magnetization of a honeycomb cluster (a), and dynamical spin structure factor (b). Panel (c) shows the interacting single particle density of states. Panel (d) shows the expected measurement in dI/dV, that combines the IETS of the spin excitations and single particle tunneling, as given by Eq. 4.

where the full many-body spectral function is

$$A_T(\omega) = \sum_\alpha A(\mathbf{r}_\alpha, \omega), \tag{3}$$

with Ω being the many-body ground state. The method of calculation of $A(\mathbf{r}_\alpha, \omega)$ is detailed later on. Overall, the full dI/dV of the system will have two contributions, the elastic and the inelastic contribution

$$\frac{dI}{dV}(\mathbf{r}_\alpha, \omega) = c_1 D(\mathbf{r}_\alpha, \omega) + c_2 \int_0^\omega A(\mathbf{r}_\alpha, \omega') d\omega'. \tag{4}$$

The elastic contribution is zero below the single particle gap, and corresponds to the local density of states we have discussed in the manuscript. The inelastic contribution $\int_0^\omega A(\mathbf{r}_\alpha, \omega')$ gives rise to steps below the single particle gap, and it is present only in the regime in which electronic correlations are dominant.

As a reference of how these two tunneling mechanisms contribute to the dI/dV, we show in Fig. S6 a typical example of how these two contributions appear on a nanographene island (Fig. S6(a)) [10], with Hamiltonian $H = t\sum_{\langle ij \rangle} c^\dagger_{i,s} c_{j,s} + U \sum_i c^\dagger_{i,\uparrow} c_{i,\uparrow} c^\dagger_{i,\downarrow} c_{i,\downarrow}$. A finite honeycomb geometry depicted has zero edge modes, which in the presence of electronic interactions gives rise to local magnetic moments (Fig. S6(a)), and opens a full single particle gap (Fig. S6(c)). However, the spin excitations remain gapless (Fig. S6(b)), and a set of

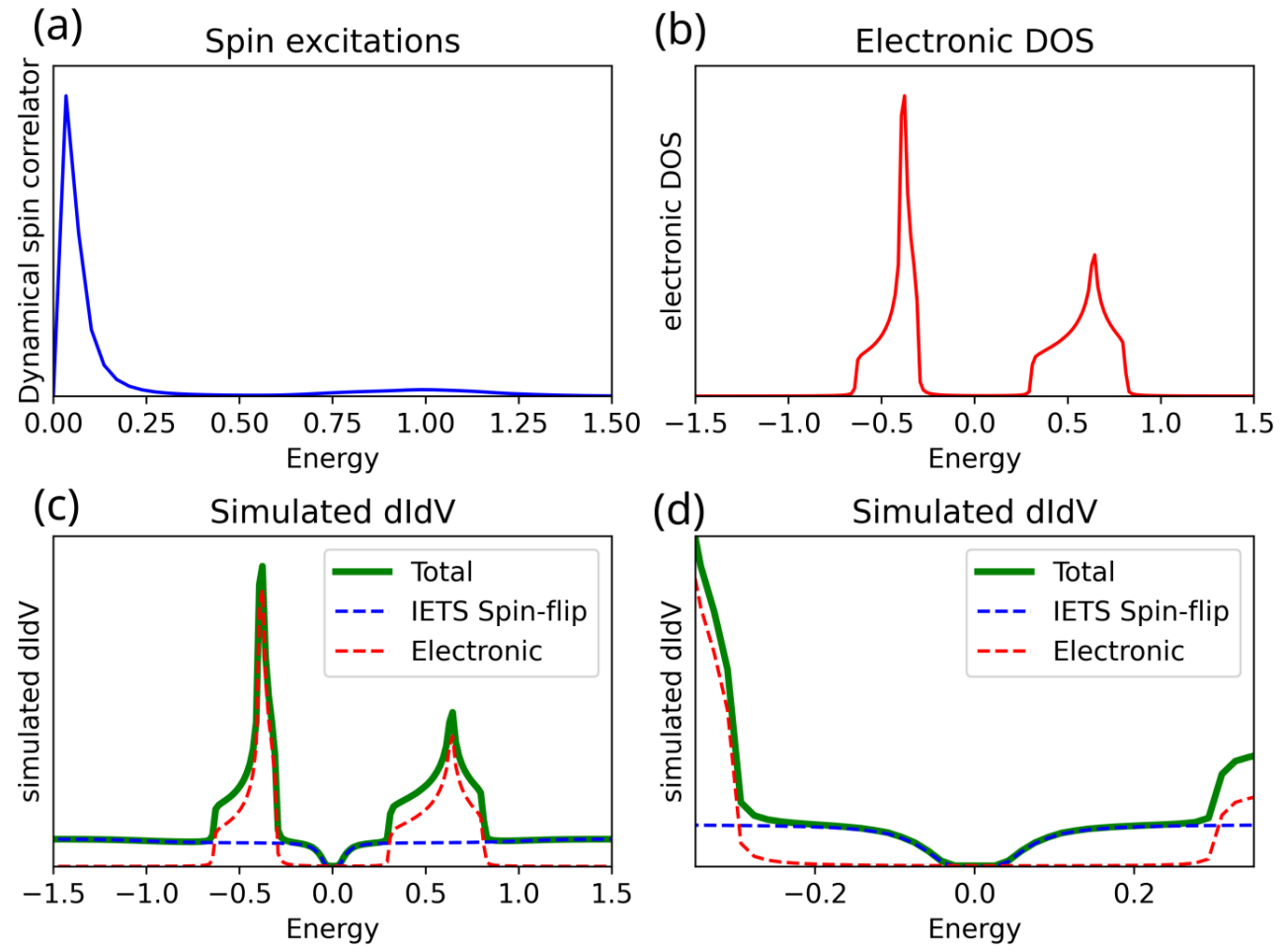

FIG. **S7**. dI/dV in a frustrated Mott monolayer. Panel (a) shows the dynamical spin structure factor, featuring low-energy excitations stemming from the local magnetic order. Panel (b) shows the density of states, featuring a gap due to electronic correlations. Panels (c,d) show the dI/dV of the system, that features steps at low energies stemming from the spin excitations, and the single particle tunneling at high energies (Eq. 4). In particular, inside the Mott gap, the signal is dominated by inelastic excitations, directly reflecting the existence of magnetic order. We took $U/t = 12$.

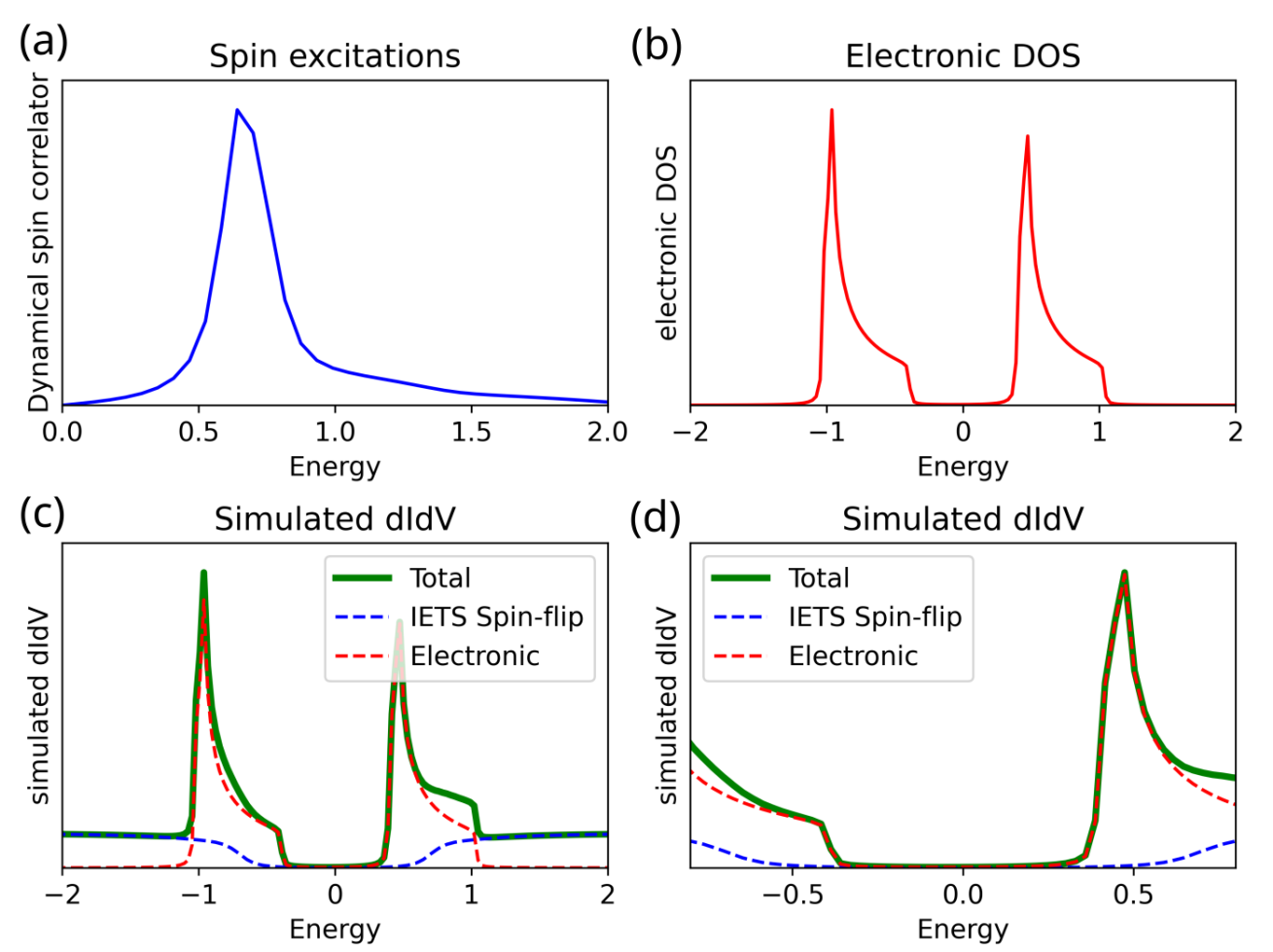

FIG. **S8**. dI/dV in a bilayer with a single particle bonding-antibonding gap. Panel (a) shows the dynamical spin structure factor, no low-energy excitations occur due to the absence of local magnetic order. Panel (b) shows the density of states, featuring a gap due to the bonding-antibonding of the layers. Panels (c,d) show the dI/dV of the system, which features a full gap at low energies without inelastic steps. Single particle tunneling and inelastic excitations only appear at high energies above the gap. The absence of low-energy inelastic steps directly reflect the absence of local order. We took $U/t = 1.4$ and $t_\perp/t = 10$

resonances appear below the electronic gap. In STM spectroscopy, this gives rise to inelastic steps below the single-particle gap in the conductance (Fig. S6(d)), which provides a direct signature of the magnetism of the system, as observed in multiple experiments [11–15].

We now discuss how the dI/dV enables seeing the presence of local magnetism in a two-dimensional system. We start by considering a minimal Mott monolayer, with a Hamiltonian of the form $H = t\sum_{\langle ij\rangle} c^\dagger_{i,s} c_{j,s} + U\sum_i c^\dagger_{i,\uparrow} c_{i,\uparrow} c^\dagger_{i,\downarrow} c_{i,\downarrow}$ in the triangular lattice. We solve the previous model in the strongly interacting regime $U/t = 12$, that gives rise to a Mott state. We show the simulated dI/dV spectroscopy in Fig. S7. Figure S7(a) shows the spin structure factor, that features low energy excitations, associated with magnons. The electronic excitations in contrast show a Mott gap, due to the strong correlations of the system (Fig. S7(b)). The simulated dI/dV includes contributions both from elastic tunneling and inelastic tunneling, as shown in Fig. S7(c,d). The inelastic contribution gives rise to steps at low energies, appearing well below the Mott gap as shown in Fig. S7(d). The presence of those subgap features directly reflect the existence of magnetic order in the Mott monolayer.

In contrast, a bilayer system whose gap stems from bonding-antibonding does feature low energy modes, as shown in Fig. S8. For the sake of concreteness, we take a bilayer triangular lattice with a Hamiltonian of the form $H = t\sum_{\langle ij\rangle,l} c^\dagger_{i,l,s} c_{j,l,s} + t_\perp \sum_{l\neq l'} c^\dagger_{i,l,s} c_{i,l,s} + U\sum_{i,l} c^\dagger_{i,l,\uparrow} c_{i,l,\uparrow} c^\dagger_{i,l,\downarrow} c_{i,l,\downarrow}$. We solve the Hamiltonian in an interacting regime but whose ground state features zero magnetization due to a bonding-antibonding single particle gap, with parameters $U/t_\perp = 1.4$, $t_\perp/t = 10$. As shown in Fig. S8(a), spin excitations no longer appear at low energies, due to the existence of a single particle gap (Fig. S8(b)) and the absence of magnetic order. The simulated dI/dV (Fig. S8(c,d)) does not show features at low energies below the electronic gap, in stark contrast with the Mott regime of Fig. S7.

With the previous results in mind, we now analyze the spin excitation of the twisted TaS_2 system. We show in Fig. S9(a) the total spin excitation spectra of the twisted system. It is observed that a peak at low energies appears, which corresponds to the magnetic excitations of the regions of the moiré featuring a Mott state. The full density of states of the system is shown in Fig. S9(b), where it is observed that the system features a full gap. Experimentally, the combination of spin excitations at low energies and a gap density of states gives rise to the dI/dV spectra shown in Fig. S9(c). The step at low energies arises from the spin excitations, whereas the high-energy peaks from the gapped electronic spectra. The spatially resolved spin excitations, which correspond to the spatially resolved d^2I/dV^2 map, are shown in Fig. S9(d), where it is observed that a finite inelastic signal appears just in a region of the moiré. In contrast, the map of the local density of states (Fig. S9(e)) shows

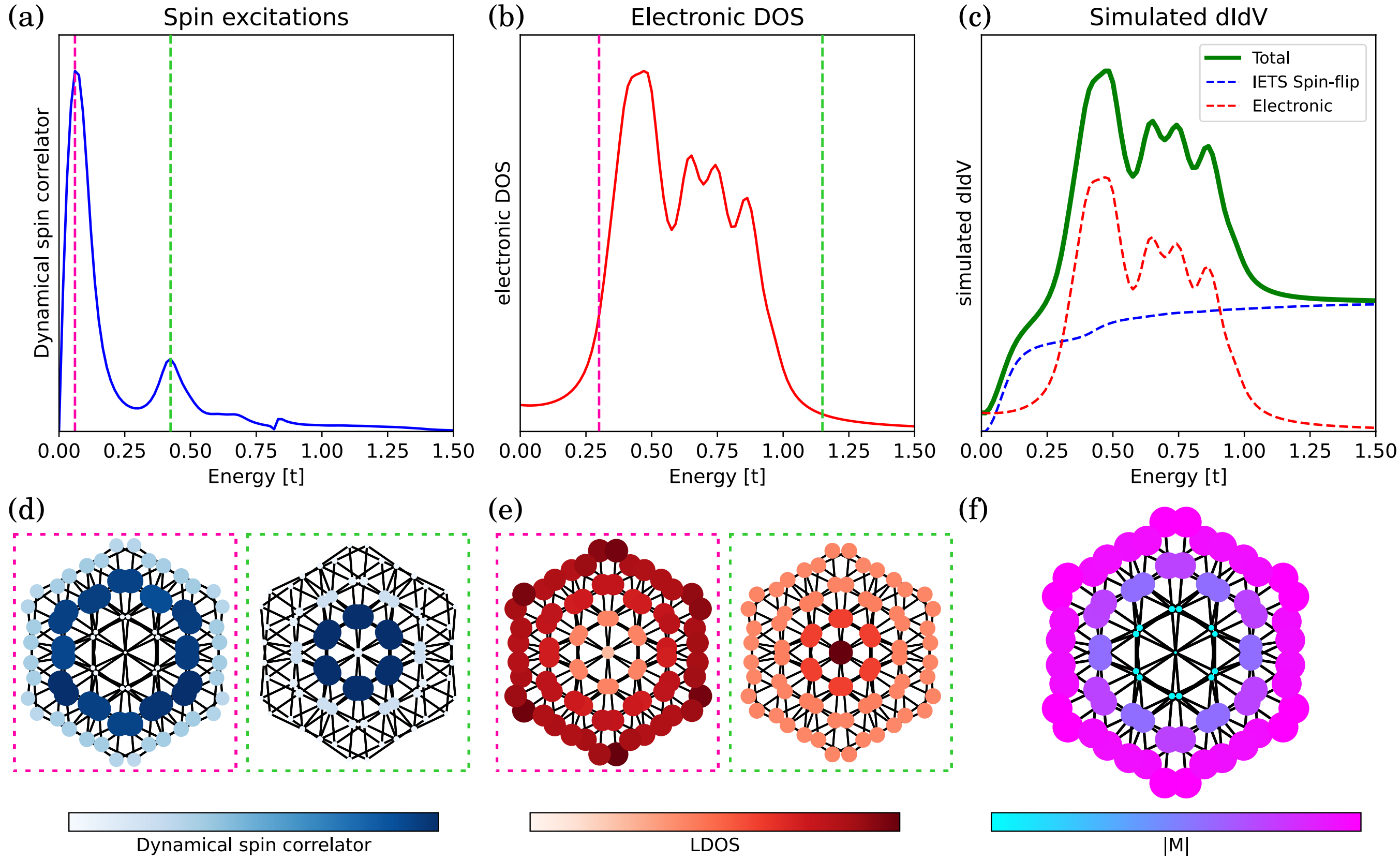

FIG. **S9**. Experimental signature of Mott gap in a twisted bilayer. Panel (a) shows the spin flip inelastic excitations (b) the single particle density of states and (c) the simulated dI/dV including both contributions. The spin excitations at low energy appear in specific regions of the moiré (d), in contrast with the local density of states spread to all regions. The magnetization arising form the Mott state (f), is located precisely in the regions where the low energy IETS is finite (d), providing a direct experimental signature of the local Mott state.

features in the whole moiré. The system develops local magnetization stemming from the Mott gap in certain regions (Fig. S9(f)), which corresponds precisely to those where low energy IETS is observed (Fig. S9(d)). The previous results directly show that the d^2I/dV^2 spectra at low energy directly reflect the spatial distribution of the Mott state, and enable identifying the Mott regions experimentally.

Calculation of spin excitations

We now elaborate on how the spin excitations can be computed. Within the mean-field approximation of our manuscript, the spin susceptibility tensor χ^{spin}_{RPA} can be computed by including electronic interactions through a random phase approximation. The interacting response function enables computing the spatially resolved dynamical spin correlator $A(\mathbf{r}_\alpha,\omega)$ as

$$A(\mathbf{r}_\alpha,\omega) = -\sum_\sigma \int \text{Im}[\chi^{\text{spin}}_{RPA}(\mathbf{q},\omega)]_{\alpha,\sigma,\alpha,\sigma} d^2\mathbf{q}, \quad (5)$$

and the total spin correlator as

$$A_T(\omega) = -\sum_{\sigma',\alpha} \int \text{Im}[\chi^{\text{spin}}_{RPA}(\mathbf{q},\omega)]_{\alpha,\sigma,\alpha,\sigma} d^2\mathbf{q}. \quad (6)$$

The interacting spin response function χ^{spin}_{RPA} can be computed by summing an infinite geometric series of bubble diagrams involving the spin susceptibility[16–18], taking the form

$$\chi^{\text{spin}}_{RPA}(\mathbf{q},\omega) = \chi^{\text{spin}}_0(\mathbf{q},\omega)[\mathcal{I} - U\chi^{\text{spin}}_0(\mathbf{q},\omega)]^{-1}. \quad (7)$$

The tensor χ^{spin}_0 is the bare spin response function of the mean-field state, which takes the form

$$\chi^{\text{spin}}_0(\mathbf{q},\omega)_{\alpha,\sigma,\beta,\sigma'} = \sum_{\mu\nu} \int d^2\mathbf{k} F_{\mu\nu}(\mathbf{k},\mathbf{q}) g^{\alpha\sigma\beta\sigma'}_{\mu\nu}(\mathbf{k},\mathbf{q}), \quad (8)$$

where

$$g^{\alpha\sigma\beta\sigma'}_{\mu\nu}(\mathbf{k},\mathbf{q}) = \frac{\langle\psi_{\mu,\mathbf{k}}|S^\sigma_\alpha|\psi_{\nu,\mathbf{k}+\mathbf{q}}\rangle\langle\psi_{\nu,\mathbf{k}+\mathbf{q}}|S^{\sigma'}_\beta|\psi_{\mu,\mathbf{k}}\rangle}{\epsilon_\mu(\mathbf{k}) - \epsilon_\nu(\mathbf{k}+\mathbf{q}) + \omega + i0^+}, \quad (9)$$

with $F_{\mu\nu}(\mathbf{k},\mathbf{q}) = f(\epsilon_\nu(\mathbf{k})) - f(\epsilon_\mu(\mathbf{k}+\mathbf{q}))$ and with $f(\epsilon)$ the Fermi-Dirac distribution. The wavefunctions ψ_α are the single particle mean-field variation wavefunctions and S^σ_α are the spin $\sigma = (x, y, z)$ operators in site α. Eq. 7 is solved by inversion of the $3N \times 3N$ χ_0^{spin} tensor in Eq. 7, with N the number of sites.

It is worth noting the key spectral difference between χ_0^{spin} and χ_{RPA}^{spin}. In the case of the bare susceptibility χ_0^{spin}, the imaginary part of the susceptibility, stemming from divergencies in Eq. 8, which is the one accounting for the spin excitations, only becomes non-zero at energies above the single particle gap as observed in Eq. 8. In stark contrast, the full spin susceptibility χ_{RPA}^{spin} can yield finite excitations at energies below the single particle gap, due to divergencies in Eq. 7 stemming from the denominator $\mathcal{I} - U\chi_0^{\text{spin}}$. In particular, in a Mott insulator, the full spin susceptibility gives rise to spin excitations well below the single particle gap, which are associated with the magnons of the magnetic state. In contrast, in a bonding-antibonding insulator, the spin excitations will not be present at low energies, as both χ_0^{spin} and χ_{RPA}^{spin} will reflect electron-hole excitations.

[1] Yonghao Liu, Yuan Zheng, Kun Yang, Wenhao Zhang, Zongxiu Wu, Jingjing Gao, Xuan Luo, Yuping Sun, Jin Zhang, and Yi Yin, "Modulation of electronic structure via dual moiré patterns in twisted 1T-TaSe$_2$," Proceedings of the National Academy of Sciences **123**, e2520703123 (2026), https://www.pnas.org/doi/pdf/10.1073/pnas.2520703123.

[2] Ying Fei, Zongxiu Wu, Wenhao Zhang, and Yi Yin, "Understanding the Mott insulating state in 1T-TaS2 and 1T-TaSe2," AAPPS Bulletin **32**, 20 (2022).

[3] Adolfo O Fumega and Jose L Lado, "Moiré-driven multiferroic order in twisted CrCl$_3$, CrBr$_3$ and CrI$_3$ bilayers," 2D Materials **10**, 025026 (2023).

[4] Tiago V. C. Antão, Jose L. Lado, and Adolfo O. Fumega, "Electric field control of moiré skyrmion phases in twisted multiferroic NiI$_2$ bilayers," Nano Letters **24**, 15767–15773 (2024).

[5] Fengrui Yao, Volodymyr Multian, Zhe Wang, Nicolas Ubrig, Jérémie Teyssier, Fan Wu, Enrico Giannini, Marco Gibertini, Ignacio Gutiérrez-Lezama, and Alberto F. Morpurgo, "Multiple antiferromagnetic phases and magnetic anisotropy in exfoliated CrBr$_3$ multilayers," Nature Communications **14** (2023), 10.1038/s41467-023-40723-x.

[6] Daniel Bennett, Gabriel Martínez-Carracedo, Xu He, Jaime Ferrer, Philippe Ghosez, Riccardo Comin, and Efthimios Kaxiras, "Stacking-engineered ferroelectricity and multiferroic order in van der Waals magnets," Phys. Rev. Lett. **133**, 246703 (2024).

[7] Muhammad Akram, Jesse Kapeghian, Jyotirish Das, Roser Valentí, Antia S. Botana, and Onur Erten, "Theory of moiré magnetism in twisted bilayer α-RuCl$_3$," Nano Letters **24**, 890–896 (2024).

[8] Fengrui Yao, Dario Rossi, Ivo A. Gabrovski, Volodymyr Multian, Nelson Hua, Kenji Watanabe, Takashi Taniguchi, Marco Gibertini, Ignacio Gutiérrez-Lezama, Louk Rademaker, and Alberto F. Morpurgo, "Moiré magnetism in CrBr$_3$ multilayers emerging from differential strain," Nature Communications **15** (2024), 10.1038/s41467-024-54870-2.

[9] J. Fernández-Rossier, "Theory of single-spin inelastic tunneling spectroscopy," Phys. Rev. Lett. **102**, 256802 (2009).

[10] Jian Zhang, Bhaskar Ghawri, Debopriya Dutta, Roman Fasel, Michel Calame, Gabriela Borin Barin, and Mickael L. Perrin, "Bottom–up-synthesized graphene nanoribbons for nanoelectronics," Nature Reviews Materials **11**, 194–212 (2026).

[11] Oliver Gröning, Shiyong Wang, Xuelin Yao, Carlo A. Pignedoli, Gabriela Borin Barin, Colin Daniels, Andrew Cupo, Vincent Meunier, Xinliang Feng, Akimitsu Narita, Klaus Müllen, Pascal Ruffieux, and Roman Fasel, "Engineering of robust topological quantum phases in graphene nanoribbons," Nature **560**, 209–213 (2018).

[12] Elia Turco, Fupeng Wu, Gonçalo Catarina, Nils Krane, Ji Ma, Roman Fasel, Xinliang Feng, and Pascal Ruffieux, "Magnetic excitations in ferromagnetically coupled spin-1 nanographenes," Angewandte Chemie International Edition **63** (2024), 10.1002/anie.202412353.

[13] Chenxiao Zhao, Lin Yang, João C. G. Henriques, Mar Ferri-Cortés, Gonçalo Catarina, Carlo A. Pignedoli, Ji Ma, Xinliang Feng, Pascal Ruffieux, Joaquín Fernández-Rossier, and Roman Fasel, "Spin excitations in nanographene-based antiferromagnetic spin-1/2 heisenberg chains," Nature Materials **24**, 722–727 (2025).

[14] Shantanu Mishra, Gonçalo Catarina, Fupeng Wu, Ricardo Ortiz, David Jacob, Kristjan Eimre, Ji Ma, Carlo A. Pignedoli, Xinliang Feng, Pascal Ruffieux, Joaquín Fernández-Rossier, and Roman Fasel, "Observation of fractional edge excitations in nanographene spin chains," Nature **598**, 287–292 (2021).

[15] Greta Lupi, Saketh Ravuri, Chenxiao Zhao, Weidan Zhang, Cesare Roncaglia, Renxiang Liu, Xinliang Feng, Daniele Passerone, Pascal Ruffieux, Roman Fasel, Jose L. Lado, and Gonçalo Catarina, "Learning Inhomogeneous Heisenberg Hamiltonians in Nanographene Spin Chains," arXiv e-prints , arXiv:2606.29281 (2026), (accessed 2026-08-16).

[16] D. L. Mills and Pascal Lederer, "Dynamical properties of magnetic impurities in transition metals," Phys. Rev. **160**, 590–599 (1967).

[17] Pietro M. Bonetti and Walter Metzner, "Spin stiffness, spectral weight, and landau damping of magnons in metallic spiral magnets," Phys. Rev. B **105**, 134426 (2022).

[18] J. W. Negele and H. Orland, *Quantum Many-Particle Systems* (Addison-Wesley, Reading, MA, 1987).
